# Reuse of Almond Skin to Formulate a New Gluten- and Lactose-Free Bakery Product

**DOI:** 10.3390/foods13233796

**Published:** 2024-11-26

**Authors:** Lucia Francesca Vuono, Vincenzo Sicari, Antonio Mincione, Rosa Tundis, Roberta Pino, Natale Badalamenti, Maurizio Bruno, Francesco Sottile, Sonia Piacente, Luca Settanni, Monica Rosa Loizzo

**Affiliations:** 1 Department of Pharmacy, Health and Nutritional Sciences, University of Calabria, 87036 Rende, Italy; luciafrancesca.vuono@unical.it (L.F.V.); roberta.pino@unical.it (R.P.); monica_rosa.loizzo@unical.it (M.R.L.); 2 Department of Agraria, Mediterranea University of Reggio Calabria, Località Feo di Vito, 89124 Reggio Calabria, Italy; vincenzo.sicari@unirc.it (V.S.); amincione@unirc.it (A.M.); 3 Department of Biological, Chemical and Pharmaceutical Sciences and Technologies, University of Palermo, Viale Delle Scienze, Bldg. 16, 90128 Palermo, Italy; natale.badalamenti@unipa.it (N.B.); maurizio.bruno@unipa.it (M.B.); 4 NBFC (National Biodiversity Future Center), 90133 Palermo, Italy; 5 Centro Interdipartimentale di Ricerca “Riutilizzo Bio-Based Degli Scarti da Matrici Agroalimentari”, University of Palermo, 90128 Palermo, Italy; francesco.sottile@unipa.it; 6 Department of Architecture, University of Palermo, Viale Delle Scienze, Bldg. 14, 90128 Palermo, Italy; 7 Department of Pharmacy, University of Salerno, Fisciano, 84084 Salerno, Italy; piacente@unisa.it; 8 Department of Agricultural, Food and Forest Sciences, University of Palermo, Viale Delle Scienze, Bldg. 5, 90128 Palermo, Italy; luca.settanni@unipa.it

**Keywords:** *Prunus amygdalus*, almond skin, celiac disease, lactose intolerance, polyphenols, bioactivity, type 2 diabetes

## Abstract

This work aimed to propose the reuse of processing waste from the Sicilian almond (*Prunus amygdalus* Batsch.) cultivar Tuono for the formulation of a new functional baked product (muffin) that is gluten- and lactose-free. Muffins were prepared using orange juice, rice flour, extra virgin olive oil, and enriched almond skin (3% and 6% *w*/*w*). The chemical-physical parameters, total phenols, and flavonoids (TPC and TFC), as well as the biological properties of the ingredients and muffins, were evaluated. Sensory analyses were also conducted. DPPH, ABTS, β-carotene bleaching, and FRAP tests were applied to measure the antioxidant potential. Muffin extracts were also tested against α-amylase and α-glucosidase enzymes. Muffins enriched with 6% almond skin (M6) showed the highest TPC and TFC with values of 26.96 mg gallic acid equivalent (GAE)/g and 24.12 mg quercetin equivalent (QE)/g, respectively. M6 exerted a promising antioxidant activity as an inhibitor of lipid peroxidation, with an IC_50_ of 15.44 μg/mL at 30 min incubation. Moreover, muffin M6 showed a promising α-glucosidase inhibitory effect (IC_50_ of 51.82 μg/mL). Based on the obtained results and supported by sensory analysis, muffins enriched with almond skin should be proposed as a promising example of upcycling for the development of a new functional bakery product.

## 1. Introduction

Currently, the reuse of agricultural waste by the food industry has become an important research topic [1]. Fruit by-products represent a significant source of raw materials for the development of functional foods [2]. Among fruit-derived by-products, almond skin is particularly rich in phenolic compounds and fibres with prebiotic effects [3], which exert health-promoting effects [4,5]. These properties can be exploited in bakery production [1,6,7,8]. Bakery products encompass a wide range of different products, both savoury and sweet. Among sweet products, muffins are the most commonly consumed, especially for breakfast. The term “muffin” first appeared in a British magazine called *London Labour* in 1851, where muffins originated. Consumers can enjoy muffins as breakfast or snacks because they come in a variety of sizes, shapes, and flavours [9].

The demand for breakfast products for special consumer categories (celiac and lactose-intolerant) has increased over the last decade. Celiac disease is a serious health problem worldwide, a multisystemic disease that affects several organs. Individuals with this condition are more likely to have digestive problems because gluten triggers an immune response in the small intestine that causes a reduction in the ability to absorb nutrients, including lactose [10]. The only way to treat all patients with gluten-related disorders, including celiac disease, gluten sensitivity (non-celiac), and wheat allergy, is by strictly adhering to a gluten-free diet. For this reason, the global gluten-free products market size is expected to grow at a compound annual growth rate (CAGR) of 9.8% from 2023 to 2030 [11]. Lactose intolerance related to primary or secondary lactase deficiency is characterized by several symptoms, such as abdominal pain and distension, flatus, and diarrhoea. Treatment of lactose intolerance can include a lactose-reduced diet and enzyme replacement [12]. The global lactose-free dairy market is estimated to grow at a CAGR of 8% from 2022 to 2031 [13]. 

Now, metabolic syndrome (MetS) represents one of the most widespread health problems in the world. This pathological condition is characterized by various metabolic anomalies, including obesity, insulin resistance, dyslipidaemia, and increased oxidative stress, which lead to an increase in the pro-inflammatory state, ultimately contributing to poor health outcomes. MetS affects almost 30% of the world’s population [14].

Several studies have shown that patients with celiac disease are at a high risk of developing MetS and fatty liver, which increases further with a gluten-free diet [15]. Moreover, patients on a gluten-free diet frequently claim lactose intolerance and therefore avoid milk and dairy products [16]. Nowadays, functional foods are used in the prevention and amelioration of several chronic diseases, including metabolic syndrome. In fact, some bioactive compounds present in functional foods can reduce dyslipidemia, improve insulin sensitivity and antioxidant status, exert anti-inflammatory activity, and facilitate body weight control [17]. Moreover, recent studies have investigated the possibility of reusing fruits, vegetables, and grain by-product ingredients to produce a nutrient-rich and highly valuable muffin [9].

In our continuous search for the reuse of almond skin by-products, we propose the development of gluten- and lactose-free muffins enriched with almond skin from the Sicilian cultivar Tuono, able to prevent metabolic syndrome and associated pathologies. This cultivar is typical of the provinces of Syracuse, Ragusa, and Agrigento. Tuono almonds are characterized by their round shape, excellent quality, and sweet flavour, but compared to all other almond varieties, it is less rich in oil. However, it contains significant amounts of proteins, B vitamins, magnesium, iron, calcium, and fibre [4]. For this purpose, functional muffins were prepared using orange juice instead of milk, rice flour instead of wheat flour, and extra virgin olive oil instead of butter. The baked products were enriched with two different percentages of almond skin (3 and 6% *w*/*w*, M3 and M6). The control muffins (MC) (muffins without the addition of almond skin) and both enriched muffins were screened for their physicochemical parameters, total phenolic and flavonoid content, and potential bioactivity to counteract metabolic syndrome and obesity. The in vitro antioxidant activity was evaluated using a multi-target approach (FRAP, ABTS, DPPH, and β-carotene bleaching tests). The inhibitory activities of the muffins on carbohydrate hydrolyzing enzymes (α-amylase and α-glucosidase) were also assessed. Moreover, to evaluate consumer acceptance, colour, texture, and sensory analyses were performed.

## 2. Materials and Methods

### 2.1. Chemicals and Reagents

Solvents used in this study were obtained from VWR International S.r.l. (Milan, Italy). Gallic acid, caffeic acid, chlorogenic acid, *p*-coumaric acid, ferulic acid, ellagic acid, quercetin, catechin, rutin, ascorbic acid, propyl gallate, butylated hydroxytoluene (BHT), β-carotene, linoleic acid, tween 20, sodium potassium tartrate, sodium chloride, sodium carbonate, ferric chloride (Fecl_3_), Folin-Ciocalteu reagent, 2,2-diphenyl-1-picrylhydrazyl (DPPH), 2,4,6-tripyridyl-s-triazine (TPTZ), 2,2′-azino-bis(3-ethylbenzothiazoline-6-sulfonic acid) diammonium salt, (ABTS) solution, buffer phosphate, starch from potato, α-amylase enzyme, α-glucosidase enzyme, dihydrochloride, maltose, potassium chlorate, aluminium chloride, sodium acetate, *o*-dianisidine (DIAN) solution, peroxidase/glucose oxidase (PGO) system-colour reagent, and dimethyl sulfoxide (DMSO) were purchased from Sigma-Aldrich S.r.l. (Milan, Italy). 

### 2.2. Muffin Preparation

**Figure 1:**
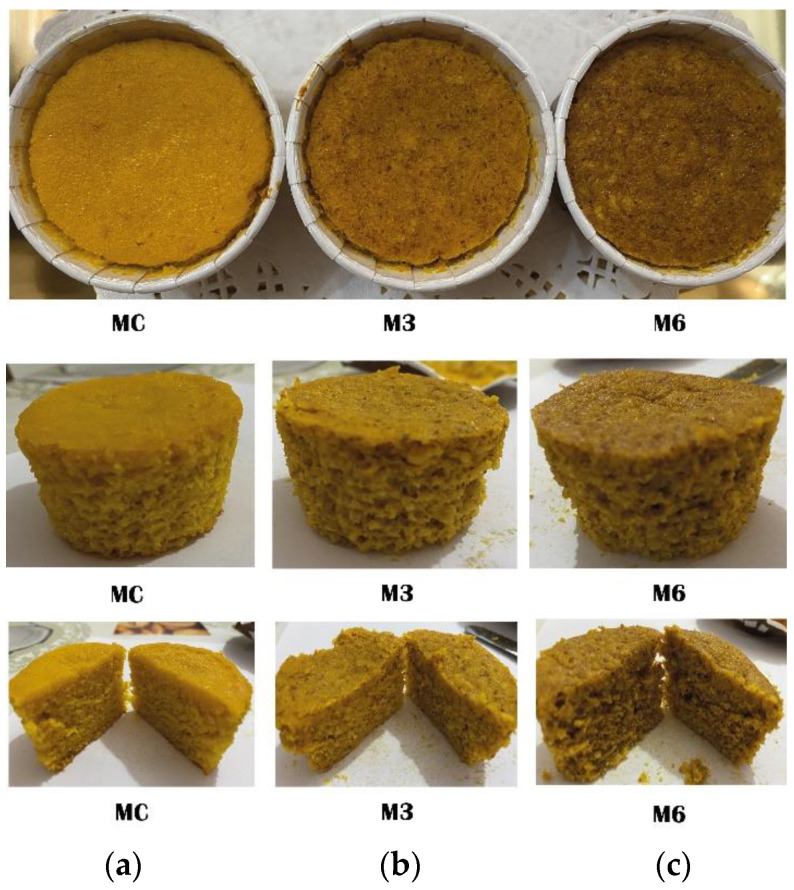
Muffins enriched with increasing levels of almond skin. From left to right: MC: control muffin prepared without adding almond skin (**a**); M3: muffin prepared by adding 3% almond skin in the dough (**b**); M6: muffin prepared by adding 6% almond skin in the dough (**c**).

Muffins were prepared using *Prunus amygdalus* Batsch cv Tuono (Sicily, Italy), orange juice variety Tarocco (Corigliano-Rossano, Italy), extra virgin olive oil (EVOO) cv Dolce di Rossano (Corigliano-Rossano, Italy), rice flour (Molino Spadoni, Conad SC, Bologna, Italy), baking powder (E450 sodium diphosphate, E500 sodium acid carbonate), vanilla flavour (Cameo S.p.a, Desenzano del Garda, Italy), Eucalyptus honey (Corigliano-Rossano, Italy), carrots, and eggs (local market, Corigliano-Rossano, Italy) (Table 1). Two levels of almond skin addition were considered: 3% (M3) and 6% (M6) on a rice flour basis, which were compared with the control muffins (MC) prepared without adding almond skin (Figure 1).

The process consisted of two steps:

1. Crush the carrots in a blender (mod. LM 871 Perfect Mix + Moulinex, Milan, Italy) for 4 min, then add orange juice and extra virgin olive oil to the blender according to Table 1.
2. Using electric whisks (mod. SuperMix 180 Moulinex, Milan, Italy), combine the eggs, honey, and almonds that were previously ground into flour using the blender, and gradually add the puree from Step 1.

Finally, yeast and vanilla aroma were added until a homogeneous dough was formed. The muffins were baked in a single-use mould in a static oven (mod. Bertazzoni, La Germania, Ravenna, Italy) at 150–160 °C for about 20 min, and then left to cool. About 20 muffins of 50 g each were obtained. Muffins were finely crushed for analysis, except for the textural, colorimetric, and sensory analyses.

### 2.3. Determination of Energy Value

To calculate the energy value of the muffins, we added the energy values of proteins, carbohydrates, and fats for each ingredient present in the sample, as reported on the label, taking into account the added quantity. The following conversion values were used: protein, 4 calories per gram; carbohydrates, 4 calories per gram; fats, 9 calories per gram [18,19].

### 2.4. Chemical-Physical Parameters

The weight of almond-enriched muffins was measured 2 h after baking using a PCE-BSK 310 laboratory balance digital scale (PCE-BSK 310, PCE Instruments, Capannori (LU), Italy), and volume was determined by using the method described by Garcìa et al. [20]. The muffins’ crumb moisture content was measured at 105 °C using a moisture analyser (MB, OHAUS, Zurich, Switzerland). The sugar content was measured with a digital refractometer (PR-201α, Tokyo, Japan) in the range of 0–60%, by stirring 5.0 g of sample in 50 g of distilled water for 5 min [21].

### 2.5. Colorimetric Parameters

The muffins’ colour differences were determined on their surfaces using a colorimeter (mod. CSM-4, PCE Instruments, Capannori, Italy). The lightness (L*) and colour parameters (+a*: red, −a*: green; +b*: yellow, −b*: blue) were assessed [22]. Colour was expressed by chroma index (C*) [Equation (1)] and hue angle (H) [Equation (2)], which were used to evaluate the degree of saturation/fullness and the amount of redness and yellowness:

(1) $$C*=\sqrt{{a*}^{2}+{b*}^{2}}$$

H = arctan (b*/a*)(2)

### 2.6. Texture Analysis

Texture analysis was performed on muffins at 25 °C. The samples were measured by a two-bite compression test using a rheometer (Compac-100II, Sun Scientific, Tokyo, Japan). For the measurement, a cylindrical probe (20 mm diameter) was mounted and operated at 1.0 mm/s. The test was carried out on the muffin mid-section, and the experiment was repeated six times. Hardness (N), springiness (%), cohesiveness (%), chewiness (g), and brittleness (g) were determined. Hardness refers to the maximum force at the maximum peak of the first compression. Springiness is the deformation rate between the first compression and the second compression, defined as the ratio of distances (d1: the maximum distance of the first bite; d2: the distance to the deformed muffin surface during the second bite). Cohesiveness is the strength of internal bonds and is defined as the ratio of areas (A). Chewiness is calculated by multiplying the hardness value by the cohesiveness value. Fracturability is a measure of force at the first peak.

Springiness (%) = d_2_/d_1_ × 100

Cohesiveness (%) = A_2_/A_1_ × 100

### 2.7. Sensory Analysis

The muffin sensory analysis was carried out at the sensory laboratory of the Department of Agraria of the Mediterranean University of Reggio Calabria using descriptive quantitative analysis. A trained panel of 10 adults was recruited among regular consumers of muffins and almonds, composed of 3 males and 7 females, aged between 22 and 25 years. Panel members followed ISO 8586:2012 training for expert sensory assessors’ selection and training. The study did not reveal any personal information or images of participants and was conducted in accordance with the Declaration of Helsinki. Panellists gave their informed consent for inclusion before they participated in the study and declared no allergies or intolerances to the sample ingredients. The descriptors used in the sensory testing were selected among those listed in [23].

The analysis was performed in sensory booths, with samples served in random order at room temperature. The panellists cleansed their palates between samples with natural mineral water. Results were expressed as the mean of a 10-point structured scale ranging from 0 (absence) to 9 (extremely high) for visual, olfactory, taste, and textural descriptors for each muffin type.

### 2.8. Extraction from Raw Materials and Muffins

The raw materials (almond, carrot, and rice flour) and the three formulations of the muffins were extracted using ultrasound-assisted extraction (3 cycles, frequency 40 kHz) for 30 min at 40 °C using a hydro-alcoholic ethanol solution (EtOH:H_2_O, 9:1) in a 1:1 ratio (sample:solvent) using a water bath (3800-CPXH; Branson, Milan, Italy). The obtained extracts were filtered through a 0.45 mm Millipore filter (GMF Whatman, Carlo Erba, Milan, Italy), and the solvent was removed. Extracts were preserved at 4 °C before subsequent analysis. 

### 2.9. Total Phenolic Content (TPC) and Total Flavonoid Content (TFC)

The total phenolic content (TPC) was determined using the Folin-Ciocalteu method, as previously described [24]. The extract (1.5 mg/mL) was mixed with Folin-Ciocalteu reagent, sodium carbonate 15% (*w*/*v*), and distilled water. After 120 min of incubation at 25 °C, the absorbance was read at 765 nm using a UV–vis Jenway 6003 spectrophotometer (Milan, Italy). Analyses were carried out in triplicate, and results were reported as mg of gallic acid equivalent (GAE)/100 g of fresh weight (FW). For the evaluation of total flavonoid content (TFC), the extract (1.5 mg/mL) was added to sodium nitrite 5% (*w*/*v*) and distilled water. After 5 min, aluminium chloride 10% (*w*/*v*) was added. After another 5 min, sodium hydroxide 1 M and water were added. Then, the absorbance was measured at 510 nm using a UV–vis Jenway 6003 spectrophotometer. Analyses were carried out in triplicate, and results were reported as mg quercetin equivalent (QE)/100 g of fresh weight (FW) [24].

### 2.10. In Vitro Antioxidant Activity

#### 2.10.1. FRAP Assay

Ferric Reducing Ability Power (FRAP) test was assessed following the procedure previously described [25]. Briefly, for the FRAP solution, tripyridyltriazine (TPTZ) reagent, FeCl_3_, HCl, and acetate buffer were mixed and then the reagent (2 mL) was diluted with distilled water (900 µL). The diluted FRAP solution was mixed with extract (1.25 mg/mL). After half an hour of incubation at room temperature, the absorbance was measured at 595 nm using the UV–Vis Jenway 6003 spectrophotometer. Butylated hydroxytoluene (BHT) was used as a positive control. The FRAP value was expressed as µM Fe (II)/g. 

#### 2.10.2. *β*-Carotene Bleaching Test

The protection from lipid peroxidation was assessed using the β-carotene bleaching test [25]. Briefly, β-carotene, linoleic acid, and Tween 20 were prepared and mixed to obtain an emulsion. Extracts at concentrations in the range of 2.5–100 µg/mL were mixed with emulsion and left to react for 30 and 60 min. The absorbance was read at 470 nm using the UV-Vis Jenway 6003 spectrophotometer (Milan, Italy) against a blank. Propyl gallate was the positive control. Results are reported as IC_50_ (half maximal inhibitory concentration) values in µg/mL.

#### 2.10.3. Evaluation of Radical Scavenging Potential by DPPH and ABTS Tests

The 2,2-diphenyl-1-picrylhydrazyl (DPPH) test was performed as previously described [25]. Briefly, the DPPH solution (1.0 × 10^−4^ M) and the extracts at different concentrations (62.5–1000 µg/mL) were mixed. After half an hour, the absorbance was read at 517 nm using a UV–vis Jenway 6003 spectrophotometer. For the ABTS (2,2′-azino-bis (3-ethylbenzothiazoline-6-sulfonic acid) diammonium salt) test, the ABTS^·+^ radical cation solution was prepared by mixing potassium persulphate (2.45 mM) and ABTS (7 mM) [25]. After 12 h, the solution was diluted with ethanol to reach an absorbance value of 734 nm. The obtained ABTS solution (2 mL) was added to the extract (25 µL) at different concentrations (1–400 µg/mL) and left to react for 6 min. The absorbance was read at 734 nm. Ascorbic acid was used as a positive control in both tests. Results are expressed as IC_50_ values (µg/mL). 

### 2.11. Carbohydrate Hydrolyzing Enzyme Inhibitory Activity

α-Amylase and α-glucosidase are enzymes involved in carbohydrate digestion and, for this reason, they have been recognized as therapeutic targets for the modulation of post-prandial hyperglycaemia [26]. To assess the α-amylase inhibitory activity, the protocol previously published was applied [26]. Briefly, samples were dissolved in methanol at different concentrations (25–1000 µg/mL) and mixed with starch and porcine pancreatic enzyme solution. After incubation at room temperature for 5 min, the absorbance was read at 540 nm. 

For the α-glucosidase inhibitory activity test, the procedure described by Leporini et al. [27] was applied. Briefly, samples at different concentrations (25–1000 µg/mL), maltose solution, and enzyme solution were mixed and left to react at 37 °C for half an hour. Perchloric acid was then added to block the enzymatic reaction. The mixture was centrifuged to collect the supernatant. The supernatant was mixed with *o*-dianisidine (DIAN) solution and peroxidase/glucose oxidase (PGO) system-colour reagent solution and left to react at 37 °C for half an hour. The absorbance was read at 500 nm.

Acarbose was used as a positive control in both tests.

### 2.12. Statistical Analysis

Linear regression, assessment of repeatability, calculation of averages, relative standard deviation (SD), and Pearson’s correlation coefficient (*r*) were calculated using Microsoft Excel 2010 software (Redmond, WA, USA). The results were expressed as means ± SD (*n* = 3). The inhibitory concentration of 50% (IC_50_) was calculated by using GraphPad Prism version 4.0 for Windows (San Diego, CA, USA). Statistical analysis was performed using one-way analysis of variance (ANOVA) followed by Tukey’s post hoc test with SPSS software for Windows, version 22.0 (Chicago, IL, USA). Differences at * *p* < 0.05 were statistically significant, while differences at ** *p* < 0.01 were highly significant. PCA and Pearson correlation analysis was carried out using SPSS software for Windows, version 22.0 (Chicago, IL, USA).

## 3. Results and Discussion

### 3.1. Nutritional Characteristics 

Table 2 shows the energy values of the control muffins (MC), and enriched muffins with 3% and 6% *w*/*w* almond skin (M3 and M6, respectively), whereas Table S1 (Supplementary Materials) reports the energy values of the ingredients used for muffin preparation.

The product enriched with almond skin was characterized by a slight increase in caloric value (100.27 and 100.61 kcal for M3 and M6, respectively) compared to the MC (99.76 kcal). Nevertheless, the differences observed in the total caloric value of the muffins are not statistically significant, nor are the differences found in carbohydrate, fat, protein, and fibre contents. Our muffins are less caloric than those formulated with grape pomace powder, where both carbohydrates and proteins are higher (40.04 and 9.72 g/100 g) [28]. For a balanced breakfast product, the ideal caloric intake varies between 150 and 200 kcal. The fibres are nutritious foods that promote metabolic well-being, as in our developed product.

### 3.2. Physical Characteristic and Sensory Profile 

The results on the weight, volume, and textural properties of enriched muffins are presented in Table 3. The addition of almond skin by-products significantly affected the physical and textural properties of the samples. 

At a 3% concentration, the weights of almond-skin-enriched muffins were significantly (*p* < 0.05) higher than those of the control muffins (50.23 vs. 53.09 g for MC and M6, respectively). This weight increase may be due to the higher fibre content of almond-skin-enriched muffins, which improved moisture retention during baking [29]. 

A significant variation in chewiness parameters was observed among the investigated samples, with values in the range 14,955.23–21,812.68 g. Previously, Na et al. [30] showed that fibre content influenced the chewiness of tiger nut dietary fibre-based muffins. This aspect can be considered positive, as it guarantees a prolonged period of chewing food before swallowing, offering a greater feeling of satiety. A different textural behaviour was observed by Demirkan et al. [31] in hazelnut-shell-based muffins. In this case, hardness and chewiness significantly decreased, while springiness increased as the amount of hazelnut shell increased in the muffin recipe.

As expected, the addition of different concentrations of almond skin to muffins caused a reduction in the chroma C* value, although no significant differences were found between control and functional muffins regarding the lightness parameter (L*) (Table 4). 

The colour changes could be due to the original colour of the raw materials used, which may result in a darker brown colour in the final baked product. Product formulation is also a critical factor for browning development. The accumulation of coloured compounds during baking is influenced by different factors such as sugar content, water activity, and processing temperature. 

Purlis et al. [32] and Sangnark and Noomhorm [33] noted that the addition of fibres to the bread darkens the colour of the final product, whereas the addition of protein can speed up the Maillard reaction, resulting in a browner colour of the crust due to the greater presence of melanoidins. As shown in Table 4, the highest brightness was observed in the crust of the control sample (MC). High brightness was also observed by Guedes de Melo et al. [34], who added chia seed to wheat flour, resulting in a more heterogeneous dough. Conversely, the substitution of wheat flour with PD-SC (a mixture of polydextrose and sucralose) in muffin recipes produced a decrease in crumb lightness [35]. A significant reduction in hue angle was observed when almond skin was added to the formulation, with values of 3.15, 2.82, and 2.49° for MC, M3, and M6, respectively.

### 3.3. Sensory Analysis

Panel results were processed and rendered in graphical form using spider plots. 

**Figure 2:**
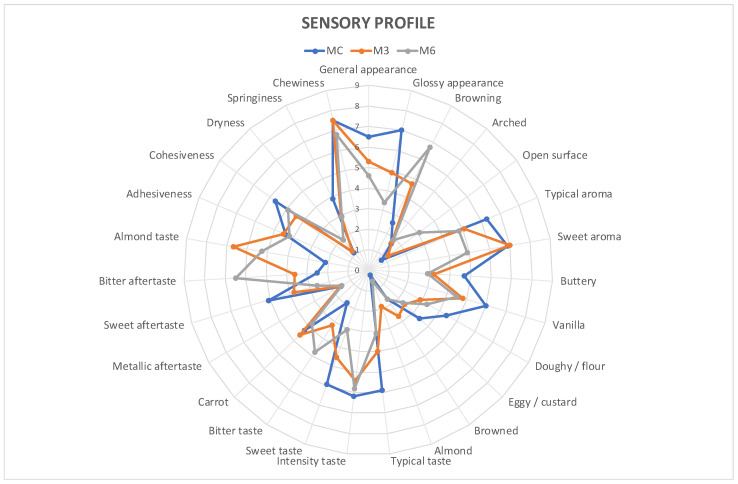
Sensory profile spider plot: MC: control muffins; M3: muffins enriched with 3% almond skin; and M6: muffins enriched with 6% almond skin.

Sensory data show the general sensory profile (Figure 2), as well as visual (Figure S1), olfactory (Figure S2), taste (Figure S3), and texture sensory profiles (Figure S4). 

From the general sensory profile, the main descriptors identified by the judges were found to be glossy appearance, typical aroma, sweet aroma, typical taste, intensity taste, sweet taste, almond taste, and chewiness (Figure 2). The modified samples (M3 and M6) showed significantly higher sensory performance than the control (MC), with an increase in the content of almond skin affecting the descriptors browning, bitter taste, aftertaste, and almond taste, while a reduction in sweetness was observed. For other descriptors, no significant variations were found. Regarding sensory differences between samples M3 and M6, significant differences were found for sample M6, which showed a lower glossy appearance, a higher degree of browning and an open surface (as a consequence of the effect of the addition of tegument in the cooking phase), lower sweet and almond aroma, and higher bitter taste and aftertaste compared to sample M3. Structural texture sensory descriptors did not show significant differences among samples, except for a slight cohesiveness and springiness decrease in the modified samples (Figure S4).

### 3.4. Bioactive Compounds in Muffin Ingredients and Baked Products

Several bioactive compounds were quantified in the muffin ingredients and baked products. The increase in TPC and TFC in the muffins functionalized with almond skin is related to EVOO. In fact, as highlighted in Table S2, the TPC and TFC values of this ingredient are the highest among all the ingredients and are equal to 708.87 mg GAE/100 g and 242.60 mg QE/100 g, respectively. Previously, Cuffaro et al. [36] investigated the TPC of EVOO from Moraiolo, Frantoio, and Leccino cultivars and found a TPC of 300 mg GA eq/Kg oil, whereas values from 460 to 778 mg/kg were found for Ciciarello and Ottobratica Cannavà, respectively, collected in October. It is interesting to note that in the same study, the TPC range observed in oil obtained in November production was 334–695 mg/kg for Ottobratica and Ottobratica Cannavà [37].

The Dolce di Rossano EVOO extract showed higher TPC and TFC values of 708.87 mg GAE/kg and 242.60 mg QE/kg, respectively, when compared to another Calabrian EVOO cultivar Carolea (TPC of 418.51 mg GAE/kg) [38]. Moreover, the contribution of EVOO’s TPC is not only related to its health-promoting properties but also its role in the formation of the typical taste of EVOO, which is recognized in muffins. A significant contribution to the content of bioactive compounds comes from the addition of Eucalyptus honey, used as a sugar substitute, as reported in the literature [39]. In fact, for EH, TPC and TFC values of 108.56 mg GAE/100 g, and 62.49 mg QE/100 g, respectively, were observed. These values are in line with those found by Alcoléa et al. [40], who reported a TPC in the range of 20.49–101.44 mg GAE/100 g and a TFC range of 1.41–13.52 mg QE/100 g in monofloral and multifloral honey produced in different regions of Brazil. 

A great variability in the TPC and TFC contents of almonds was documented by Ozcan et al. [41], who found values of TPC between 0.7 and 7.5 mg GAE/100 g, and TFC between 2.43 and 19.49 mg QE/100 g. 

As shown in Table S2, the almond extract in this study showed a TFC of 21.35 mg QE/100 g. This value is in line with the study by Loizzo et al. [4], in which a TFC of 20.38 mg QE/100 g was found in ethanol extracts of the Italian almond cv Casteltermini. 

Previously, lower TPC values in the range from 9.9 to 26.8 mg GAE/100 g were reported for Californian almond skins from Fritz, Buttle, Price, and Padre cultivars by Milbury et al. [42]. 

A high TPC was found in the Portuguese almond cv Duro Italiano (163.71 mg GAE/100 g) by Barreira et al. [43]. Moreover, as previously reported by Loizzo et al. [4] and Garofalo et al. [8], almond skin is rich in dietary fibres such as lignin, cellulose, and hemicellulose that can act on microbiota as prebiotics. 

The red Tarocco variety of orange juice from Calabria showed TPC and TFC values of 70.02 mg GAE/100 g and 35.28 mg QE/100 g, respectively. These values are lower than those reported by Legua et al. [44], who evaluated three different genotypes of Tarocco oranges, Comune, Rosso, and Messina, and found TPC values of 117.26, 236.34, and 241.91 mg GAE/L.

TFC values from 17.58 to 18.23 mg QE/100 mL of juice were found in another typical Calabrian *Citrus C. clementina* juice [27]. Considering the high TPC and TFC contents of the individual ingredients, the effects of the muffin formulation and cooking process were also evaluated by measuring the bioactive content in control and almond-enriched muffins.

**Figure 3:**
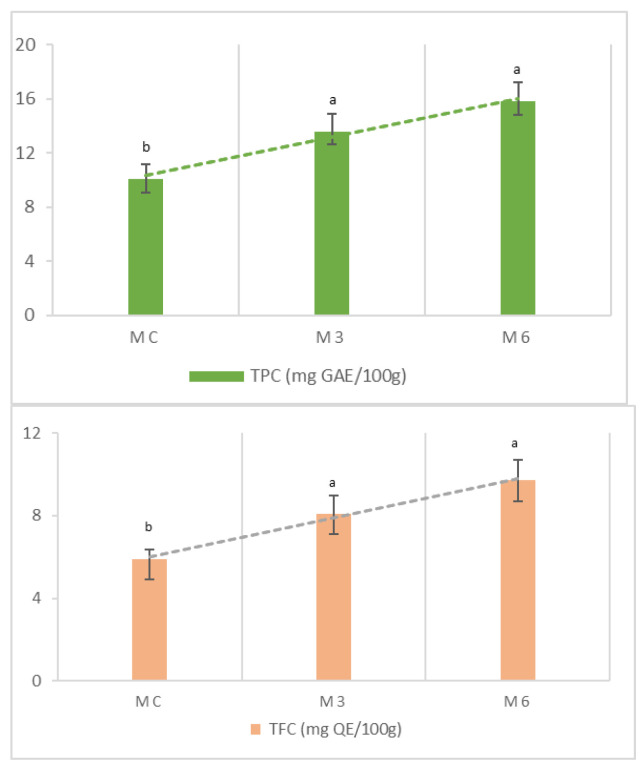
Total phenol content (TPC) and total flavonoid content (TFC). MC: control muffins; M3: muffins enriched with 3% almond skin; M6: muffins enriched with 6% almond skin. Differences between samples were evaluated by one-way ANOVA followed by Tukey’s test. Results followed by different letters in each column are significantly different.

As shown in Figure 3, the TPC and TFC are proportional to the amount of almond skin added to the dough formulation (3% and 6%). The sample enriched with 6% almond skin (*w*/*w*) (M6) showed the highest TPC and TFC, with values of 15.8 mg GAE/100 g and 9.7 mg QE/100 g, respectively, compared to the control muffins. 

Previously, Loizzo et al. [4] reported the formulation of functional jam obtained by adding from 10 to 20% (*w*/*w*) of almond tegument to blackberry jam. The highest TPC and TFC content was found in jam enriched with 20% almond tegument, with values of 26.96 mg CAE/g and 24.12 mg QE/g, respectively. On the other hand, Pasqualone et al. [1] evaluated three formulations of biscuits enriched with different percentages of almond peel. The authors found that the formulation with 20% of tegument contained about twice the TPC compared to the control, indicating that the addition of almond peel to the formulation can contribute to improving the nutritional value and potential health benefits of the final baked product. Similarly, Nakow et al. [45] found a significant increase in TPC in biscuits enriched with 6% grape pomace compared to the control. Confirming this, the results of the study by Olawuyi et al. [46] demonstrated that the incorporation of mushrooms (MP) and carrots (CP) into muffins increased the TPC compared to the control rice muffins. 

Lower values of TPC were found in rice muffins enriched with wheat, corn, oat, and barley flours, ranging from 675 to 1687 μg/g [21]. Previously, Aranibar et al. [47] proposed the reutilization of chia oil extraction residue in wheat muffins. The addition of this residue increased the TPC in a dose-dependent manner, as well as antioxidant activities. More recently, Giri et al. [48] formulated muffins enriched with pomegranate peel (PP), a by-product of the pomegranate processing industry, and found an increase in TPC values from 0.443 to 48.53 mg GAE/100 g for 8% PP-muffins compared to control muffins. These bakery products demonstrated antioxidant features in the same order of magnitude as the products proposed in this research [49]. 

### 3.5. Antioxidant Activity

The antioxidant activity of enriched muffins was assessed using a multi-target approach based on the application of different in vitro tests, including the DPPH test, ABTS test, FRAP assay, and β-carotene bleaching test. As shown in Table S3, among the ingredients used for muffin preparation, Dolce di Rossano EVOO extract showed the highest FRAP value, which was better than the positive control BHT (FRAP values of 90.63 and 63.26 μM Fe (II)/g), respectively). 

The PR sample exhibited weak FRAP activity with a value of 25.76 μM Fe (II)/g. This result is consistent with those reported for Casteltermini peel ethanol extract (FRAP value of 21.38 μM Fe (II)/g) by Loizzo et al. [4]. However, these values are higher than those reported by Ozcan et al. [41] (FRAP values of 0.04 and 0.96 μM Fe (II)/g). 

In the β-carotene bleaching test, the extract obtained from the almond skin (AS) showed the highest lipid peroxidation protective effect, with IC_50_ values of 9.09 and 13.19 μg/mL at t = 30 and 60 min, respectively. These values are consistent with those found for the almond cv Casteltermini [4]. 

As previously mentioned, the blanching process causes a reduction in the content of bioactive compounds responsible for antioxidant activity, such as vitamins and flavonoids. Moreover, biodegradation as a consequence of high-temperature processing may also occur. On the other hand, the drying and roasting of the rice can produce a series of reactions that can affect the concentration of phenolic compounds and result in the production of new compounds, such as melanoidins, which exhibit antioxidant properties [50].

Almond flour showed promising ABTS radical scavenging potential, with an IC_50_ value comparable to the positive control ascorbic acid (2.98 and 1.72 μg/mL, respectively).

Radical scavenging assessed by DPPH revealed that Tuono almond flour (PR) has greater radical scavenging potential than the Duro Italiano cultivar, with IC_50_ values of 31.22 and 800 μg/mL, respectively [43].

EVOO showed promising radical scavenging activity, with IC_50_ values of 26.3 and 29.2 μg/mL in ABTS and DPPH, respectively. A similar ABTS activity was reported by Sicari et al. [51] in screenings of EVOO Calabrian cultivars. Among them, Coratina EVOO extract showed the highest ABTS radical scavenging potency, with an IC_50_ value of 21.37 μg/mL, followed by Ottobratica EVOO extract (IC_50_ value of 26.33 μg/mL). This trend was also observed in the DPPH test, where IC_50_ values of 29.23 and 35.82 μg/mL were found for Ottobratica and Coratina EVOO extracts, respectively. 

The Dolce di Rossano EVOO used in our recipes also demonstrated the ability to protect from lipid peroxidation (a lipid bilayer model of the cell membrane), with an IC_50_ value of 56.3 μg/mL. Moreover, the EVOO extract exhibited ferric reducing ability power (FRAP) comparable to the positive control (BHT), with a FRAP value of 90.6 μM Fe (II)/g. The addition of almond skin to the muffin formulation resulted in a significant increase in iron reduction activity, with a maximum FRAP value observed in M6 (FRAP value of 45.13 μM Fe (II)/g). The same was also found in the β-carotene bleaching test, with IC_50_ values of 19.78 and 15.44 g/mL for M3 and M6, respectively. These values were significantly lower than those for the muffin control (IC_50_ of 94.16 g/mL). At the same time, the behaviour of the muffin extracts towards the DPPH and ABTS radicals was not consistent. In fact, the DPPH radical was more sensitive, with an IC_50_ value 9.2 times lower in M6 than in the control. In contrast, Demirkan et al. [31] observed no antioxidant activity against the DPPH radical in hazelnut shell (HS)-enriched muffins compared to the control muffins, regardless of the concentration (*p* > 0.05). Low antioxidant activity was found against the ABTS radical, with IC_50_ values between 22.74 and 30.45% for M6 and MC at 400 g/mL (Table 5). 

Pearson’s correlation coefficient revealed a positive correlation for both muffin TPC and TFC and antioxidant activity, with *r* values of 0.93, 0.97, 0.94, 0.96, and 0.92, 0.98, 0.93, 0.95, for DPPH, ABTS, and β-carotene bleaching tests at 30- and 60-min incubation, respectively. 

Previously, Garofalo et al. [8] reported the UHPLC-HRMSMS analysis of the ethanol extract of Tuono almond skin and identified flavonoids as the most abundant compounds. Among them, catechin, epicatechin, quercetin, kaempferol, isorhamnetin, eriodyctiol-glycoside, naringenin, prunin, cyanidin-glycoside, and delphinidin-glycoside were the most represented. These compounds are known in the literature to be among the most powerful phytochemicals with antioxidant activity. Their mechanism of action is based on hydrogen atom transfer to free radicals. Accordingly, the more the flavonoid structure makes hydrogen transfer faster and easier, the greater its antioxidant power. Therefore, the antioxidant activity of flavonoids with hydroxyl groups in their structure is the highest among different flavonoids [52].

Pasqualone et al. [1] reported the antioxidant activity of cookies enriched with almond tegument and found a dose-dependent relationship. In fact, in biscuits enriched with 20 g of tegument per 100 g of wheat flour (AS20), the antioxidant activity is about five times higher than in the control. The same evidence was found by Loizzo et al. [4]. Indeed, the increase in antioxidant activity was proportional to the amount of almond tegument added to the formulation. Specifically, the antioxidant potential was significantly higher in jam enriched with 20% almond tegument (*w*/*w*), with increases of +20, and +21% in DPPH and ABTS activity, respectively, compared to unenriched jam extract (EM). Moreover, an increase in iron reduction capacity of 8% compared to the control was observed in the same sample. 

On the contrary, a decrease in both DPPH and ABTS radical scavenging activity was observed by Guedes de Melo et al. [34], who evaluated the antioxidant activity of muffins enriched with 6% and 12% chia seeds (M-CHIA6 and M-CHIA12, respectively). The inhibitions were 39.72 and 42.56% in DPPH, and 88.50 and 65.59% in ABTS for M-CHIA6 and M-CHIA12, respectively.

Previously, Radünz et al. [53] investigated the antioxidant activity of gluten-free green banana flour muffins and found better DPPH radical scavenging activity, with a percentage inhibition of 65.58% vs. 54.9% for control muffins. 

In another study, Rico et al. [54] investigated the effect of temperature, cereal base (rice/corn), and pulse concentration on extruded flour properties and antioxidant activity. The use of corn in combination with lentils resulted in improved antioxidant properties in the baked products. In particular, antioxidant capacity was high in 15% extruded-lentil flour muffins.

### 3.6. Effect on Carbohydrate-Hydrolyzing Enzymes

The growing interest of consumers in functional foods that can prevent obesity and related diseases, such as type 2 diabetes and MetS, is a topic of great interest for the food industry (Table S4 and Table 6).

Among the raw materials used in the formulation of the muffins, Tarocco orange juice was found to be most active in inhibiting α-glucosidase, with an IC_50_ value of 50.76 μg/mL, followed by rice flour extract (FR). The α-amylase enzyme was more sensitive to the extract obtained from the almond skin (AS) than other samples, with an IC_50_ of 75.89 μg/mL. This value is similar to that of the Casteltermini variety, with an IC_50_ value of 73.76 μg/mL [4]. 

The α-amylase and α-glucosidase inhibitory activities of Tarocco juice align with those found in *Citrus clementina* juice (IC_50_ of 67.19 and 139.89 μg/mL, respectively) [27]. The α-amylase and α-glucosidase inhibitory activity was also observed with Dolce di Rossano EVOO extract, with IC_50_ values of 62.2 and 71.5 μg/mL, respectively. In particular, the activity against the α-amylase enzyme is comparable to acarbose, a widely prescribed drug for the treatment of type 2 diabetes. The enrichment of muffins with Tuono almond skin significantly increased the inhibitory activity against the α-glucosidase enzyme, with an IC_50_ value of 51.82 μg/mL for M6 compared to the control muffins (MC, IC_50_ of 221.40 μg/mL) (Table 6). 

A similar trend was observed against α-amylase, although in this case the samples, even if enriched, showed weak activity. For M6, activity was 7.75 times lower than that of acarbose, used as the positive control. At the same time, the M3 sample showed a selectivity index (SI) against α-amylse of 11.89. 

Pearson’s correlation coefficient revealed a positive correlation between muffin TPC and TFC and α-glucosidase activity, with *r* values of 0.95 and 0.93, respectively.

Enrichment positively impacted the inhibitory activity against enzymes involved in carbohydrate digestion, with increases of +18 and 21% in inhibitory activity against α-amylase and α-glucosidase, respectively, in blackberry jam enriched by 20% almond tegument [4]. Moreover, Tsujita et al. [55] hypothesized that almond polymerized polyphenols are responsible for the α-amylase inhibitory activity, with consequent delay in the absorption of carbohydrates. 

The fortification of muffins with 2% to 6% brewer’s spent grain protein hydrolysates enhanced bioactivity. The α-amylase and α-glucosidase inhibition increased from 6% to 88.87% and 40.81%, respectively [56]. More recently, Gu et al. [57] identified two α-glucosidase inhibitory peptides from almond oil manufacturing residue. The IC_50_ of Trp-His was 17.03 μM before and after digestion, whereas the IC_50_ of Trp-Ser significantly increased after simulated digestion, from 24.71 to 44.63 μM. This study confirms that almond processing waste is still rich in bioactive compounds. Notably, Farazi et al. [58] demonstrated the potent inhibitory activity of almond peel polyphenolic extracts on human salivary α-amylase. Many studies are conducted on porcine pancreatic a-amylase, which has a sequence homology of approximately 86% with the corresponding human enzyme, although with some amino acid variations located inside the enzyme’s active site. For this reason, the type of experimental model applied in our study is preferable to that of microbial derivation for facilitating reliable predictions of effects in intervention studies.

### 3.7. Principal Component Analysis

Principal component analysis (PCA) is a dimensional reduction technique with applications in exploratory data analysis, visualization, and data pre-processing.

**Figure 4:**
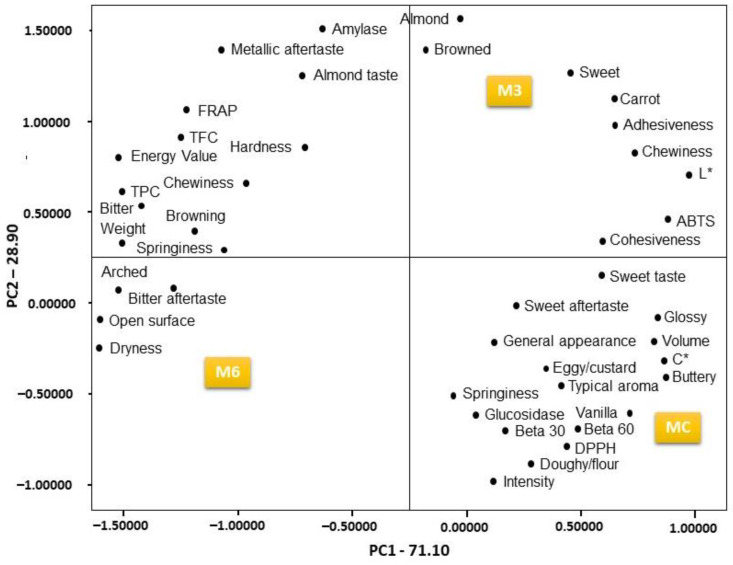
Principal component analysis of the control muffins and muffins enriched with almond tegument: projections of the samples (MC, M3, M6) and their physical, chemical, and sensory profiles.

Figure 4 shows the PCA bi-dimensional map of control (MC) and muffins with added almond tegument (M3 and M6), along with their corresponding analytical profile. The first two principal components explain 100% of the total variance. The PCA was performed on the overall set of average values, with 71.10% in the first principal component (PC1) and 28.90% in the second (PC2) (Table S5). Figure 4 shows a clear separation between control and enriched muffins, characterized by positive and negative values in components 1 and 2, respectively.

In this study, a Pearson correlation test was performed to evaluate the relationships between the different parameters analysed (Table S6). A high correlation was observed between the FRAP and weight, hardness, chewiness, springiness, TPC, TFC, α-amylase, embrowning, arched surface, bitterness, metallic aftertaste, bitter aftertaste, and almond taste. Other correlations were observed between BETA 30 (β-carotene bleaching test after 30 min of incubation) and BETA 60 (β-carotene bleaching test after 60 min of incubation) with cohesiveness, DPPH, ABTS, α-glucosidase, L*, C*, general appearance, glossy surface, typical aroma, buttery flavour, vanilla, doughy/floury texture, eggy/custard-like notes, typical taste, intensity, sweetness, and sweet aftertaste. Further relationships were found between α-amylase and TFC, TPC, FRAP, browning, almond, metallic aftertaste, and almond taste, and ABTS with BETA 30, BETA 60, DPPH, α-glucosidase, L*, C*, general appearance, glossy surface, typical aroma, sweet flavour, buttery notes, vanilla, doughy/floury texture, eggy/custard-like notes, typical taste, sweet taste, sweet aftertaste, adhesiveness, dryness, and springiness.

PC1 showed a positive correlation with volume, cohesiveness, BETA 30, BETA 60, DPPH, ABTS, glucosidase, L*, C*, general appearance, glossy appearance, typical aroma, sweetness, buttery notes, vanilla, doughy/floury texture, eggy/custard notes, typical taste, sweet taste, carrot flavour, sweet aftertaste, adhesiveness, springiness, and chewiness. In addition, from the analysis of variable loads, PC1 showed a negative correlation with weight, energy value, TPC, TFC, FRAP, α-amylase, browning, arched surface, open surface, bitterness, metallic aftertaste, bitter aftertaste, almond taste, and dryness.

PC2 was correlated with TFC, FRAP, α-amylase, sweetness, browning, almond, carrot flavour, metallic aftertaste, almond taste, and adhesiveness.

Muffins enriched with 3% almond tegument (M3) were placed in the quadrant characterized by the highest overall quality scores (also higher than MC and M6).

## 4. Conclusions

Consumer preferences have changed in recent years, with consumers increasingly becoming aware of the importance of a diet in improving their quality of life and preventing chronic degenerative diseases. Several bakery products have been formulated by using functional ingredients able to exert positive effects on human health, providing a competitive advantage over traditional products in the market. In this context, the present work studied, for the first time, the enrichment of a gluten- and lactose-free baked product (muffin) with almond skin. Muffins enriched with almond skin showed increased levels of bioactive compounds, along with enhanced antioxidant and inhibitory activity of the enzymes involved in carbohydrate digestion, principally α-glucosidase.

The addition of almond processing waste impacted several important sensory appearance descriptors, such as browning, and modified samples showed increases in the bitterness and almond taste descriptors, with reductions in sweetness descriptors.

Therefore, the use of almond skin as an enrichment matrix for the formulation of gluten- and lactose-free muffins is a possible way to convert a low-value by-product into a valuable resource. This approach offers the almond processing industry an efficient and environmentally friendly solution for waste disposal, while capitalizing on the bioactive and health-promoting properties of almond skin. However, our results derive from in vitro studies, and further investigation using animal models will be necessary.

## Figures and Tables

**Table 1:** Muffin recipes.

Samples						Ingredients					
	FR (g)	PR (g)	SA (mL)	EVOO (g)	EH (g)		CRT (g)	E (unit)	AS (g)	BP (g)	VF (g)
MC	100	100	100	55	60		180	2	0	16	0.5
M3	100	100	100	55	60		180	2	21	16	0.5
M6	100	100	100	55	60		180	2	42	16	0.5

Quantities refer to 900 g of dough. MC: control muffins; M3: muffins enriched with 3% almond skin; M6: muffins enriched with 6% almond skin. FR: rice flour; PR: *Prunus amygdalus* cv Tuono flour; SA: orange juice; EVOO: extra virgin olive oil cultivar Dolce di Rossano; EH: Eucalyptus honey; AS: almond skin; CRT: carrot; E: egg; BP: baking powder and VF: vanilla flour.

**Table 2:** Macronutrients and energy values (kcal) of the muffins (~50 g of each, *n* = 3).

Macronutrients	Sample			
	MC	M3	M6	Sign.
Carbohydrates (g)	9.71 ± 0.82 ^a^	9.76 ± 0.91 ^a^	9.79 ± 0.85 ^a^	ns
Fats (g)	5.88 ± 0.43 ^b^	5.91 ± 0.48 ^a^	5.93 ± 0.56 ^a^	**
Protein (g)	2.00 ± 0.12 ^a^	2.01 ± 0.14 ^a^	2.02 ± 0.13 ^a^	ns
Fibre (g)	0.53 ± 0.07 ^b^	0.55 ± 0.08 ^ab^	0.58 ± 0.09 ^a^	*
Energy value (kcal)	99.76 ± 4.23 ^a^	100.27 ± 4.81 ^b^	100.61 ± 4.92 ^c^	**

MC: control muffins; M3: muffins enriched with 3% almond skin; M6: muffins enriched with 6% almond skin. Differences between samples were evaluated by one-way ANOVA followed by Tukey’s test. Results followed by different letters in each column are significantly different at ** *p* < 0.01, * *p* < 0.05.

**Table 3:** Muffin physical parameters.

Samples	Weight (g)	Volume (cm^3^)	Hardness (g/cm)	Chewiness (g)	Cohesiveness (%)	Springiness (%)
MC	50.23 ± 7.32 ^c^	204.81 ± 7.80 ^a^	9887.55 ± 123.40 ^b^	14955.23 ± 176.83 ^c^	120.18 ± 4.78 ^b^	100.09 ± 2.21 ^c^
M3	51.81 ± 8.51 ^a,b^	197.34 ± 7.25 ^a,b^	11565.40 ± 201.22 ^a^	18966.75 ± 545.67 ^b^	117.22 ± 3.89 ^a^	103.61 ± 2.44 ^b^
M6	53.09 ± 9.84 ^a^	192.62 ± 6.19 ^c^	11880.22 ± 186.31 ^a^	21812.68 ± 588.19 ^a^	114.12 ± 1.76 ^a^	107.98 ± 3.57 ^a^
Sign.	**	**	**	**	**	**

MC: control muffins; M3: muffins enriched with 3% almond skin; M6: muffins enriched with 6% almond skin. Differences between samples were evaluated by one-way ANOVA followed by Tukey’s test. Results followed by different letters in each column are significantly different at ** *p* < 0.01.

**Table 4:** Chroma (C*), luminosity (L*), and hue angle values in muffin formulations.

Colorimetric Parameters	MC	M3	M6	Sign.
L*	47.66 ± 4.35 ^a^	35.16 ± 4.04 ^b^	22.49 ± 3.77 ^c^	**
C*	37.03 ± 4.15 ^a^	32.20 ± 4.09 ^b^	29.82 ± 3.78 ^c^	**
Hue	3.15 ± 0.76 ^a^	2.82 ± 0.58 ^b^	2.49 ± 0.45 ^c^	**

MC: control muffins; M3: muffins enriched with 3% almond skin; M6: muffins enriched with 6% almond skin. Differences between samples were evaluated by one-way ANOVA followed by Tukey’s test. Results followed by different letters in each row are significantly different at ** *p* < 0.01.

**Table 5:** Antioxidant activity of enriched muffins.

Sample	FRAP Test	β-Carotene Bleaching Test		DPPH Test	ABTS Test
		t = 30 min	t = 60 min		
	μM Fe (II)/g	IC_50_ (μg/mL)	IC_50_ (μg/mL)	IC_50_ (μg/mL)	Inhibition (%) at 400 mg/mL
MC	0.29 ± 0.005 ^b^	94.16 ± 5.37 ^b^	94.56 ± 5.44 ^b^	630.49 ± 10.11 ^c^	30.45 ± 5.32 ^a^
M3	42.33 ± 2.45 ^a^	19.78 ± 1.23 ^a^	64.66 ± 5.52 ^a^	85.00 ± 5.32 ^b^	27.34 ± 4.87 ^a^
M6	45.13 ± 2.78 ^a^	15.44 ± 1.23 ^a^	60.77 ± 4.67 ^a^	68.61 ± 4.05 ^a^	22.74 ± 4.09 ^b^
Sign.	**	**	**	**	**

MC: control muffins; M3: muffins enriched with 3% almond skin; M6: muffins enriched with 6% almond skin. Data are reported as mean standard deviation (SD) (*n* = 3). Ascorbic acid, BHT, and propyl gallate were used as positive controls in the antioxidant tests: ascorbic acid IC_50_ of 5.02 ± 0.79 μg/mL in the DPPH test, and 1.75 ± 0.12 μg/mL in the ABTS test; BHT 63.26 ± 2.71 μMFe (II)/g in the FRAP test; propyl gallate was used in the β-carotene bleaching test with IC_50_ of 0.09 ± 0.04, and 0.08 ± 0.06 μg/mL at t = 30 and 60 min, respectively. Differences between samples were evaluated by one-way ANOVA followed by Tukey’s test. Results followed by different letters in each column are significantly different at ** *p* < 0.01.

**Table 6:** Inhibition of enzymes (IC_50_, μg/mL) linked to type 2 diabetes by muffins.

Sample	α-Glucosidase	α-Amylase	SI
MC	221.40 ± 8.75 ^c^	39.49% ^	NC
M3	64.92 ± 4.44 ^b^	772.46 ± 5.44 ^b^	11.89
M6	51.82 ± 3.87 ^a^	275.06 ± 3.22 ^a^	5.31
Sign	**	**	

MC: control muffins; M3: muffins enriched with 3% almond skin; M6: muffins enriched with 6% almond skin. NC: not calculable. Data are reported as mean standard deviation (SD) (*n* = 3). ^: at 400 mg/mL. SI: selectivity index = α-amylase/α-glucosidase. Positive control: acarbose IC_50_ of 35.51 ± 1.10 μg/mL in the α-glucosidase test and IC_50_ of 50.12 ± 1.13 μg/mL in the α-amylase test. Differences between samples were evaluated by one-way ANOVA followed by Tukey’s test. Results followed by different letters in each column are significantly different at ** *p* < 0.01.

## Data Availability

The original contributions presented in this study are included in the article and its Supplementary Materials. No data were deposited in any publicly available repositories. Further inquiries can be directed to the corresponding authors.

## Supplementary Materials

The following supporting information can be downloaded at: https://www.mdpi.com/article/10.3390/foods13233796/s1, Figure S1. Spider plot of the appearance sensory profile for the bakery products under evaluation. Figure S2. Spider plot of the olfactory sensory profile for the bakery products under evaluation. Figure S3. Spider plot of the taste sensory profile for the bakery products under evaluation. Figure S4. Spider plot of the texture profile for the bakery products under evaluation. Table S1. Energy value (kcal) of the ingredients in three muffin formulations per 100 g. Table S2. Total phytochemical content (TPC and TFC) of muffin ingredients. Table S3. Antioxidant activity of muffin ingredients. Table S4. Inhibition of enzymes linked to type 2 diabetes by muffin ingredients. Table S5. Total variance explained in factor analysis with all predictors and two factors. Table S6. A-B-D. Correlation matrix.
