# Supplementary material for: Reuse of Almond Skin to Formulate a New Gluten- and Lactose-Free Bakery Product
Source: Foods. 2024 Nov 26;13(23):3796. doi: 10.3390/foods13233796 (PMC11639755; doi:10.3390/foods13233796)
Supplement: Supplementary file 1 [file foods-13-03796-s001.zip › foods-3314945-supplementary.pdf]

Supplementary Materials

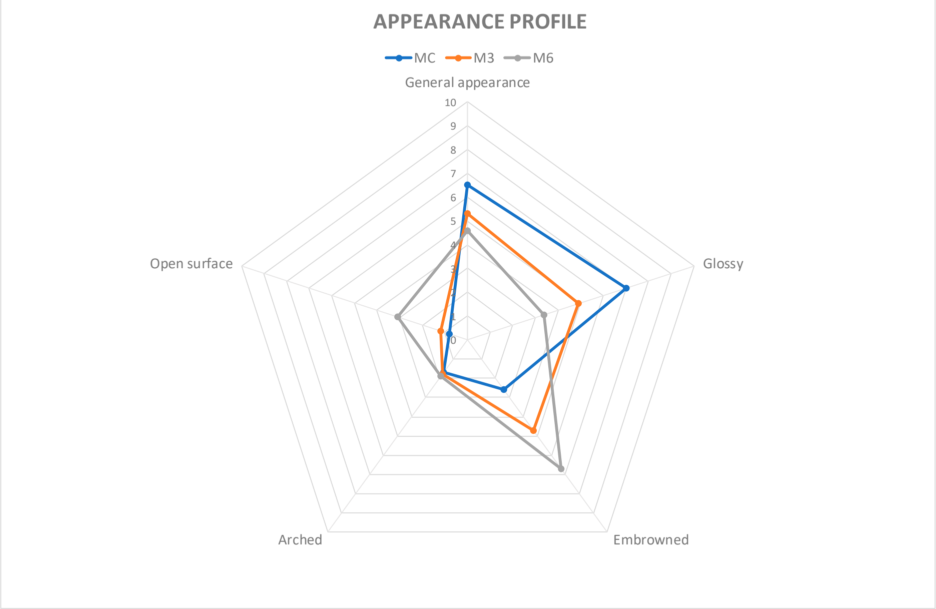

Figure S1. Appearance descriptors profile spider plot: MC: Control Muffin, M3: muffin enriched with 3% of almond skins and M6: muffin enriched with 6% of almond skins.

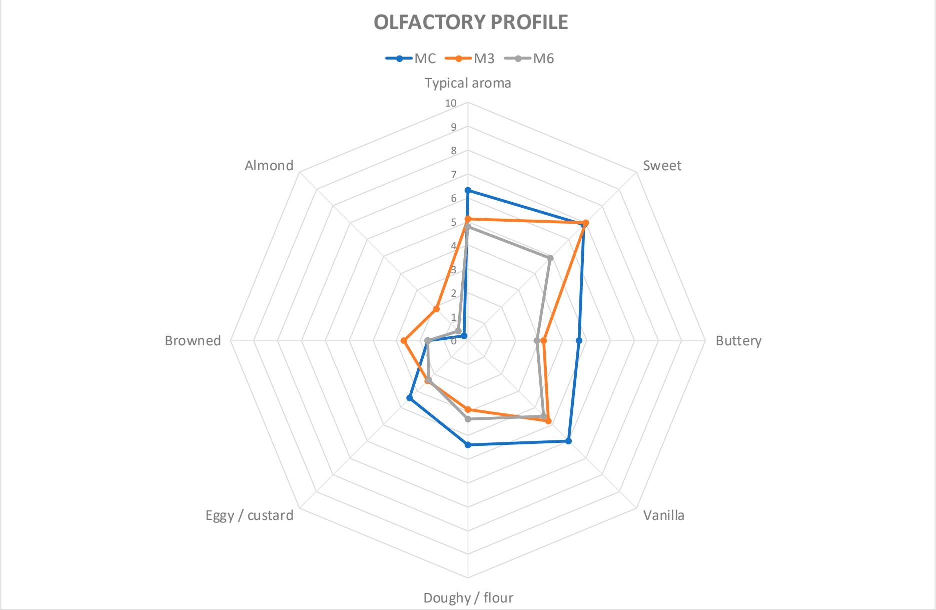

Figure S2. Olfactory descriptors profile spider plot: MC: Control Muffin, M3: muffin enriched with 3% of almond skins and M6: muffin enriched with 6% of almond skins.

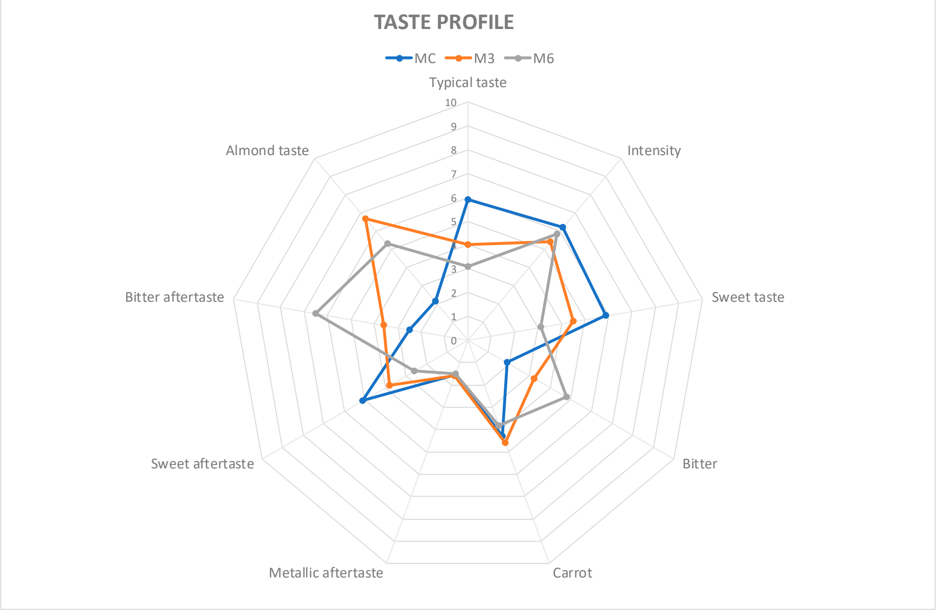

Figure S3. Taste descriptors profile spider plot: MC: Control Muffin, M3: muffin enriched with 3% of almond skins and M6: muffin enriched with 6% of almond skins.

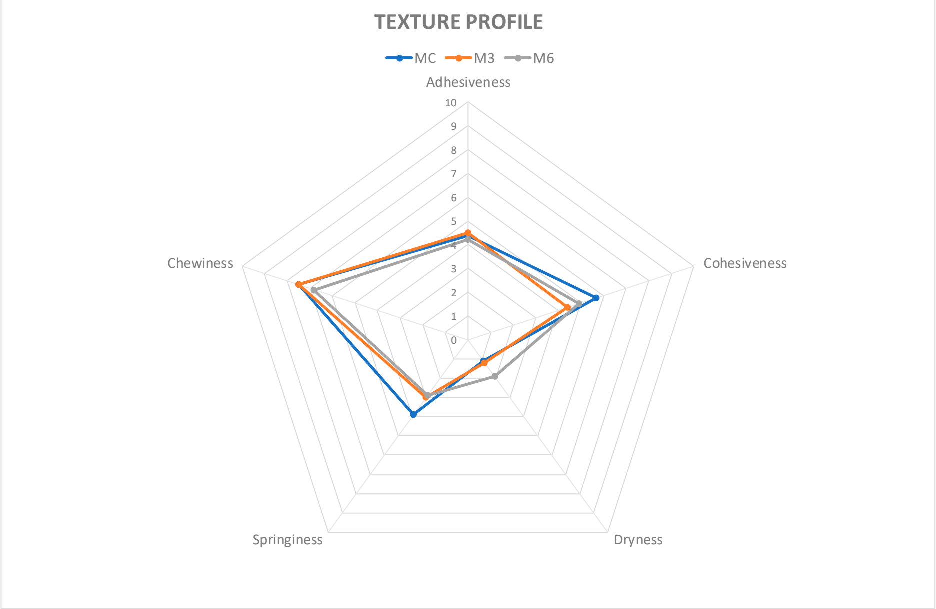

Figure S4. Sensory textural descriptors profile spider plot: MC: Control Muffin, M3: muffin enriched with 3% of almond skins and M6: muffin enriched with 6% of almond skins.

Table S1 . Energy Value (kcal) of the ingredients of three formulations of Muffin per 100g

	Sample									
	PR	AS	FR	SA	EVOO	EH	CRT	E	BP	VF
Carbohydrates (g)	8.77	3.02	86.09	10		75	18.30	0.72	66	97
Fats (g)	52.52	2.48	0.5	0.2	99.9			9.51		
Protein (g)	21.40	0,6	0.5	0.7		0.4	2.60	12.56		0.1
Fiber (g)	9.90	2.8	0.1	0.2					0.7	
Energy Value (kcal)	590	37	354	40	899	302	83	139	594	873

PR: Prunus amygdalus cv Tuono flour; AS: Almond Skin; FR: Rice flour; SA: Orange juice, EVOO: Extra virgin olive oil; EH: *Eucalyptos* Honey; CRT: Chopped carrot; E: Egg; BP: Baking Powder; VF: Vanilla Flour

Table S2. Total phytochemicals content (TPC and TFC) of muffin ingredients.

Sample	TPC (mg GAE/100 g)	TFC (mg QE/100 g)
PR	57.72 ± 3.12[c]	21.35 ± 2.72[c]
CRT	35.05 ± 2.23[e]	15.1 ± 1.08[e]
FR	6.82 ± 0.31[f]	3.84 ± 1.79[f]
EVOO	708.87±23.61[a]	242.60 ± 12.91[a]
EH	108.56 ±10.23[b]	62.49 ±7.44[b]
AS	56.68 ± 3.12 [d]	20.38 ± 2.76[d]
Sign.	**	**
SA*	70.02 ± 3.77	35.28 ± 2.15

TPC: Total Phenol Content; TFC: Total Flavonoid Content; PR: *Prunus amygdalus* cv Tuono flour; CRT: Chopped carrot; FR: Rice flour; EVOO: Extra virgin olive oil; EH: *Eucalyptus* honey; AS: Almond Skin; SA: Orange juice (*mg/100 ml); TPC: mg gallic acid equivalent (GAE)/100 g FW. TFC: mg quercetin equivalent (QE)/100 g FW; (*EVOO: mg GAE/kg and mg QE/ kg). Data are reported as mean standard deviation (SD) (n = 3). Results followed by different letters in each column are significantly different at $^{**}p < 0.01$. ns: not significant.

Table S3. Antioxidant activity of muffin ingredients.

Sample	FRAP Test	β-Carotene Bleaching Test		DPPH Test	ABTS Test
		t = 30 min	t = 60 min		
	μM Fe (II)/g	IC_{50} (μg/mL)	IC_{50} (μg/mL)	IC_{50} (μg/mL)	IC_{50} (μg/mL)
PR	25.76 ± 1.85 [c]	10.11 ± 1,06[c]	15.45 ± 1.62[d]	31.22 ± 2,14[d]	2.98 ± 0.75[c]
CRT	n.a.	34.98 %^	16.46 %^	986.56 ± 11.56[a]	49.33%^
FR	26.56 ± 1.77 [b]	44.1 ± 2.66[b]	22.03 ± 2.23[b]	345.41 ± 6.45[b]	49.25% ^
EVOO	90.63 ± 2.81[a]	56.3 ± 1.[6a]	61.33 ± 2.7[a]	29.2 ± 2.6[e]	26.3 ± 1.8[b]
AS	21.38 ± 1.74[d]	9.09 ± 1.18[d]	13.19 ± 1.41[d]	35.40 ± 2.05[c]	35.40 ± 2.05[a]
Sign.	**	**	**	**	**
SA*	0.39 ± 0.09	8.93 ± 0.65	54.71 ± 3.94	178.82 ± 4.77	27.32%^

Data are reported as mean standard deviation (SD) (n= 3). PR: *Prunus amygdalus* cv Tuono flour; 443 CRT: Chopped carrot; FR: Rice flour; EVOO: Extra Virgin Olive Oil; AS: Almond Skin; SA: Orange 444 juice (*mg/100 mL). NA: not active. ^: at 400 mg/mL. Positive controls: ascorbic acid IC_{50} of 5.02 ± 445 0.79 μg/mL in the DPPH test, and 1.75 ± 0.12 μg/mL in ABTS test; BHT 63.26 ± 2.71 μM Fe (II)/g in the FRAP test; propyl gallate in the β-carotene bleaching test IC_{50} of 0.09 ± 0.04 and 0.08 ± 0.06 447 μg/mL at t= 30 and 60 min, respectively. Differences between samples were evaluated by one-way 448 ANOVA followed by Tukey's test. Results followed by different letters in each column are significantly different at **p < 0.01.

Table S4. Inhibition of enzymes linked to type 2 diabetes by muffin ingredients.

Sample	α-Glucosidase	α-Amylase	SI[#]
PR	61.21 ± 2.88 [b]	75.89 ± 4.05 [a]	1.24
CRT	262.36 ± 7.84 [c]	469.79 ± 8.92 [c]	1.79
FR	56.29 ± 3.34 [a]	997.79 ± 13.24 [d]	17.72
SA	50.76 ± 4.06 [a]	142.06 ± 5.33 [b]	2.80
EVOO	71.50 ± 3.8	62.20 ± 2.51[ns]	0.87
AS	57.09 ± 2.57	73.76 ± 3.89	1.29
Sign	**	**	

PR: *Prunus amygdalus* cv Tuono flour; CRT: Chopped carrot; FR: Rice flour; EVOO: Extra Virgin Olive Oil; AS: Almond Skin; SA: Orange juice (*mg/100 mL). [#]SI: Selectivity Index= α-amylase/α-glucosidase. Data are reported as mean standard deviation (SD) (n = 3). Positive control: acarbose IC_{50} of 35.51 ± 1.10 μg/mL in α-glucosidase test and IC_{50} of 50.12 ± 1.13 μg/mL in α-amylase test. Differences between samples were evaluated by one-way ANOVA followed by Tukey's test. Results followed by different letters in each column are significantly different at ** p < 0.01.

Table S5. Total variance explained in factor analysis with all predictors and two factors

Component	Initial Eigenvalues			Rotation Sums of Squared Loadings		
	Total	% of Variance	Cumulative %	Total	% of Variance	Cumulative %
1	34,820	77,378	77,378	31,995	71,101	71,101
2	10,180	22,622	100,000	13,005	28,899	100,000
3	1,81E-015	4,02E-015	100,000			

Table S6. A-B-C. Correlation Matrix

A

		Weight	Volume	Hardness	Chewiness	Cohesivenes	Springiness	ENERGY VAL	TPC	TFC	FRAP	BETA 30	BETA 60
Correlation	Weight	1	-0,9976183	0,9506003	0,9993032	-0,9972762	0,9924933	0,9985167	0,9975204	0,9505711	0,9181746	-0,9154983	-0,9366994
	Volume	-0,9976183	1	-0,9697476	-0,9994977	0,9898135	-0,9816938	-0,999894	-0,999999	-0,9697246	-0,9433145	0,9410684	0,9586193
	Hardness	0,9506003	-0,9697476	1	0,9615244	-0,9251154	0,9055006	0,9660914	0,9700897	1	0,995797	-0,9951607	-0,9991147
	Chewiness	0,9993032	-0,9994977	0,9615244	1	-0,9938282	0,9872368	0,9998531	0,9994522	0,9614985	0,9323223	-0,9298772	-0,9491156
	Cohesivenes	-0,9972762	0,9898135	-0,9251154	-0,9938282	1	-0,9988104	-0,9917811	-0,9896125	-0,9250796	-0,8864527	0,8833304	0,9083229
	Springiness	0,9924933	-0,9816938	0,9055006	0,9872368	-0,9988104	1	0,9843623	0,9814252	0,9054607	0,8628302	-0,8594224	-0,8868467
	ENERGY VAL	0,9985167	-0,999894	0,9660914	0,9998531	-0,9917811	0,9843623	1	0,9998726	0,9660671	0,9383832	-0,9360454	-0,9543736
	TPC	0,9975204	-0,999999	0,9700897	0,9994522	-0,9896125	0,9814252	0,9998726	1	0,9700668	0,9437799	-0,9415427	-0,9590184
	TFC	0,9505711	-0,9697246	1	0,9614985	-0,9250796	0,9054607	0,9660671	0,9700668	1	0,9958057	-0,99517	-0,9991186
	FRAP	0,9181746	-0,9433145	0,995797	0,9323223	-0,8864527	0,8628302	0,9383832	0,9437799	0,9958057	1	-0,9999775	-0,9987685
	BETA 30	-0,9154983	0,9410684	-0,9951607	-0,9298772	0,8833304	-0,8594224	-0,9360454	-0,9415427	-0,99517	-0,9999775	1	0,9984135
	BETA 60	-0,9366994	0,9586193	-0,9991147	-0,9491156	0,9083229	-0,8868467	-0,9543736	-0,9590184	-0,9991186	-0,9987685	0,9984135	1
	DPPH	-0,9058216	0,9328865	-0,9925856	-0,9210037	0,8721062	-0,8472088	-0,9275448	-0,9333917	-0,992597	-0,999546	0,9997255	0,9968203
	ABTS	-0,9853122	0,9711869	-0,8836302	-0,9782517	0,9952234	-0,9987998	-0,9745532	-0,9708511	-0,8835861	-0,8370365	0,83335	0,8631513
	AMYLASE	0,4068794	-0,4689186	0,6703405	0,440692	-0,3383949	0,2921071	0,4560119	0,4701592	0,6704104	0,7354858	-0,7400111	-0,7009653
	GLUCOSIDAS	-0,9235208	0,9477769	-0,9969593	-0,9371934	0,8927157	-0,8696806	-0,9430338	-0,9482241	-0,9969666	-0,999906	0,9997916	0,9993549
	L*(LUMINOS	-0,9187616	0,8893408	-0,7508182	-0,9033848	0,9453795	-0,96015	-0,8959025	-0,8886975	-0,750756	-0,6871683	0,6822829	0,7223658
	C* CHROMA	-0,9911368	0,9979393	-0,9834124	-0,9954046	0,9786388	-0,9674498	-0,9968996	-0,9980285	-0,9833954	-0,9626668	0,9608306	0,974911
	General appe	-0,9959263	0,9997739	-0,9747185	-0,998598	0,9865628	-0,9774223	-0,9993585	-0,9998028	-0,9746975	-0,9501579	0,9480467	0,9644556
	Glossy	-0,999372	0,999436	-0,9610029	-0,9999982	0,9940363	-0,9875363	-0,9998189	-0,9993878	-0,9609769	-0,9316365	0,9291796	0,9485181
	Embrowned	0,9989234	-0,9933445	0,9351766	0,9964958	-0,9996241	0,9970982	0,9949159	0,9931817	0,9351433	0,8988076	-0,895849	-0,9194482
	Arched	0,9981712	-0,9916243	0,9300969	0,9952193	-0,9999111	0,9980713	0,9933993	0,9914418	0,9300623	0,8925465	-0,8895035	-0,9138206
	Open surface	0,9128437	-0,882506	0,7410031	0,8969672	-0,9404732	0,955927	0,8892585	0,8818444	0,7409398	0,6763876	-0,6714351	-0,7120969
	Typical arom	-0,9629702	0,9792732	-0,9990908	-0,9723624	0,9404616	-0,9227687	-0,976221	-0,9795568	-0,9990868	-0,990987	0,9900667	0,9964126
	Sweet	-0,564411	0,5061276	-0,2802815	-0,5332058	0,6237602	-0,6611313	-0,5186285	-0,5049153	-0,2801911	-0,1911871	0,1846032	0,2396495
	Buttery	-0,9711184	0,9852631	-0,9972105	-0,9793475	0,9508749	-0,9346482	-0,9826688	-0,9855024	-0,9972034	-0,9861831	0,9850504	0,9931874
	Vanilla	-0,9629702	0,9792732	-0,9990908	-0,9723624	0,9404616	-0,9227687	-0,976221	-0,9795568	-0,9990868	-0,990987	0,9900667	0,9964126
	Doughy / flo	-0,7494737	0,7933531	-0,9179569	-0,7736621	0,6986022	-0,6628814	-0,7844075	-0,7942077	-0,9179943	-0,9504297	0,9524927	0,9338326
	Eggy / custar	-0,9861752	0,9952562	-0,9888965	-0,9916731	0,971267	-0,9585066	-0,9937345	-0,9953919	-0,9888825	-0,9711298	0,9695088	0,9817691
	Browned	0,0604505	-0,1291563	0,3673141	0,0976655	0,0133369	-0,0620787	0,1147079	0,1305495	0,3674017	0,4509554	-0,4569283	-0,4061182
	Almond	0,2024263	-0,2694922	0,4964177	0,238838	-0,1296443	0,0811396	0,2554455	0,270845	0,4964994	0,5738367	-0,5793137	-0,5324986
	Typical taste	-0,989813	0,9972759	-0,9851118	-0,9944374	0,9766157	-0,9649705	-0,9960965	-0,9973786	-0,9850956	-0,9652262	0,9634522	0,9770072
	Intensity	-0,5514372	0,6076647	-0,7831518	-0,5821905	0,4884054	-0,4452739	-0,5960396	-0,6087801	-0,7832104	-0,8368121	0,8404633	0,808619
	Sweet taste	-0,9981712	0,9916243	-0,9300969	-0,9952193	0,9999111	-0,9980713	-0,9933993	-0,9914418	-0,9300623	-0,8925465	0,8895035	0,9138206
	Bitter	0,999923	-0,9966854	0,9466743	0,9987629	-0,9981148	0,9939348	0,997764	0,9965701	0,946644	0,9131868	-0,9104344	-0,9322815
	Carrot	-0,5699615	0,5119285	-0,2867443	-0,5388951	0,6290136	-0,6661726	-0,5243788	-0,510721	-0,2866541	-0,1977978	0,1912225	0,246187
	Metallic afte	0,5514372	-0,6076647	0,7831518	0,5821905	-0,4884054	0,4452739	0,5960396	0,6087801	0,7832104	0,8368121	-0,8404633	-0,808619
	Sweet aftert	-0,9993008	0,9943419	-0,9383295	-0,9972089	0,9993366	-0,9963719	-0,9957828	-0,9941916	-0,9382969	-0,9027202	0,8998159	0,9229534
	Bitter afterta	0,950897	-0,9272837	0,8078463	0,9386818	-0,9711355	0,9816114	0,9326348	0,9267567	0,8077908	0,75047	-0,7460229	-0,7823353
	Almond taste	0,5174152	-0,575208	0,7574901	0,5489953	-0,4528889	0,4088756	0,5632396	0,5763568	0,7575516	0,8140989	-0,8179734	-0,7842848
	Adhesivenes	-0,6077602	0,5515375	-0,331228	-0,5776958	0,6646772	-0,7003181	-0,5636215	-0,5503649	-0,3311391	-0,2434186	0,2369114	0,2912395
	Cohesivenes	-0,3349218	0,3991164	-0,6108665	-0,3698582	0,2645117	-0,2171718	-0,3857271	-0,4004043	-0,610941	-0,6808118	0,6857064	0,643634
	Dryness	0,8919641	-0,8586553	0,7075597	0,8744675	-0,9228809	0,9405606	0,8660254	0,8579343	0,7074931	0,6398653	-0,6346995	-0,6772042
	Springiness	-0,9315288	0,9543945	-0,9984004	-0,9444537	0,9021682	-0,8800598	-0,9499475	-0,954813	-0,9984057	-0,9993824	0,9991244	0,9998951
	Chewiness	-0,8342164	0,7941937	-0,6218305	-0,8130524	0,8726168	-0,8953944	-0,8029551	-0,7933391	-0,6217568	-0,5474902	0,5418685	0,5883327

B

	DPPH	ABTS	AMYLASE	GLUCOSIDAS	L*(LUMINOS	C* CHROMA	General appe	Glossy	Embrowned	Arched	Open surface	Typical arom	Sweet	Buttery	Vanilla
Weight	-0,9058216	-0,9853122	0,4068794	-0,9235208	-0,9187616	-0,9911368	-0,9959263	-0,999372	0,9989234	0,9981712	0,9128437	-0,9629702	-0,564411	-0,9711184	-0,9629702
Volume	0,9328865	0,9711869	-0,4689186	0,9477769	0,8893408	0,9979393	0,9997739	0,999436	-0,9933445	-0,9916243	-0,882506	0,9792732	0,5061276	0,9852631	0,9792732
Hardness	-0,9925856	-0,8836302	0,6703405	-0,9969593	-0,7508182	-0,9834124	-0,9747185	-0,9610029	0,9351766	0,9300969	0,7410031	-0,9990908	-0,2802815	-0,9972105	-0,9990908
Chewiness	-0,9210037	-0,9782517	0,440692	-0,9371934	-0,9033848	-0,9954046	-0,998598	-0,9999982	0,9964958	0,9952193	0,8969672	-0,9723624	-0,5332058	-0,9793475	-0,9723624
Cohesivenes	0,8721062	0,9952234	-0,3383949	0,8927157	0,9453795	0,9786388	0,9865628	0,9940363	-0,9996241	-0,9999111	-0,9404732	0,9404616	0,6237602	0,9508749	0,9404616
Springiness	-0,8472088	-0,9987998	0,2921071	-0,8696806	-0,96015	-0,9674498	-0,9774223	-0,9875363	0,9970982	0,9980713	0,955927	-0,9227687	-0,6611313	-0,9346482	-0,9227687
ENERGY VAL	-0,9275448	-0,9745532	0,4560119	-0,9430338	-0,8959025	-0,9968996	-0,9993585	-0,9998189	0,9949159	0,9933993	0,8892585	-0,976221	-0,5186285	-0,9826688	-0,976221
TPC	-0,9333917	-0,9708511	0,4701592	-0,9482241	-0,8886975	-0,9980285	-0,9998028	-0,9993878	0,9931817	0,9914418	0,8818444	-0,9795568	-0,5049153	-0,9855024	-0,9795568
TFC	-0,992597	-0,8835861	0,6704104	-0,9969666	-0,750756	-0,9833954	-0,9746975	-0,9609769	0,9351433	0,9300623	0,7409398	-0,9990868	-0,2801911	-0,9972034	-0,9990868
FRAP	-0,999546	-0,8370365	0,7354858	-0,999906	-0,6871683	-0,9626668	-0,9501579	-0,9316365	0,8988076	0,8925465	0,6763876	-0,990987	-0,1911871	-0,9861831	-0,990987
BETA 30	0,9997255	0,83335	-0,7400111	0,9997916	0,6822829	0,9608306	0,9480467	0,9291796	-0,895849	-0,8895035	-0,6714351	0,9900667	0,1846032	0,9850504	0,9900667
BETA 60	0,9968203	0,8631513	-0,7009653	0,9993549	0,7223658	0,974911	0,9644556	0,9485181	-0,9194482	-0,9138206	-0,7120969	0,9964126	0,2396495	0,9931874	0,9964126
DPPH	1	0,8201718	-0,7555652	0,9990389	0,664968	0,9540743	0,9403334	0,9202651	-0,8851929	-0,8785547	-0,6538895	0,9865011	0,1615276	0,9807443	0,9865011
ABTS	0,8201718	1	-0,2449144	0,8444604	0,9726863	0,9538941	0,9659004	0,9786424	-0,992173	-0,9938329	-0,96916	0,9027872	0,6970848	0,9161111	0,9027872
AMYLASE	-0,7555652	-0,2449144	1	-0,7261261	-0,0131709	-0,5246249	-0,4875916	-0,4389931	0,364065	0,3509149	-0,0015658	-0,6380947	0,5244265	-0,613083	-0,6380947
GLUCOSIDAS	0,9990389	0,8444604	-0,7261261	1	0,6970657	0,966288	0,9543436	0,9365318	-0,9047338	-0,8986462	-0,6864238	0,9927307	0,2046285	0,9883619	0,9927307
L*(LUMINOS	0,664968	0,9726863	-0,0131709	0,6970657	1	0,8581694	0,8794181	0,9041944	-0,9360878	-0,940948	-0,9998914	0,7782954	0,8444747	0,7980248	0,7782954
C* CHROMA	0,9540743	0,9538941	-0,5246249	0,966288	0,8581694	1	0,999078	0,9952217	-0,9839071	-0,9812936	-0,8505109	0,9902513	0,4497455	0,9942078	0,9902513
General appe	0,9403334	0,9659004	-0,4875916	0,9543436	0,8794181	0,999078	1	0,998496	-0,990671	-0,988654	-0,8723073	0,9833582	0,4876761	0,988677	0,9833582
Glossy	0,9202651	0,9786424	-0,4389931	0,9365318	0,9041944	0,9952217	0,998496	1	-0,9966522	-0,9954023	-0,897802	0,971919	0,5348053	0,9789633	0,971919
Embrowned	-0,8851929	-0,992173	0,364065	-0,9047338	-0,9360878	-0,9839071	-0,990671	-0,9966522	1	0,9999009	0,9308023	-0,9494264	-0,602098	-0,9590044	-0,9494264
Arched	-0,8785547	-0,9938329	0,3509149	-0,8986462	-0,940948	-0,9812936	-0,988654	-0,9954023	0,9999009	1	0,9358567	-0,9449112	-0,6132803	-0,9549191	-0,9449112
Open surface	-0,6538895	-0,96916	-0,0015658	-0,6864238	-0,9998914	-0,8505109	-0,8723073	-0,897802	0,9308023	0,9358567	1	-0,7689578	-0,8522758	-0,7890576	-0,7689578
Typical arom	0,9865011	0,9027872	-0,6380947	0,9927307	0,7782954	0,9902513	0,9833582	0,971919	-0,9494264	-0,9449112	-0,7689578	1	0,3209513	0,999486	1
Sweet	0,1615276	0,6970848	0,5244265	0,2046285	0,8444747	0,4497455	0,4876761	0,5348053	-0,602098	-0,6132803	-0,8522758	0,3209513	1	0,3511488	0,3209513
Buttery	0,9807443	0,9161111	-0,613083	0,9883619	0,7980248	0,9942078	0,988677	0,9789633	-0,9590044	-0,9549191	-0,7890576	0,999486	0,3511488	1	0,999486
Vanilla	0,9865011	0,9027872	-0,6380947	0,9927307	0,7782954	0,9902513	0,9833582	0,971919	-0,9494264	-0,9449112	-0,7689578	1	0,3209513	0,999486	1
Doughy / flo	0,9593664	0,6254147	-0,9097016	0,9460766	0,4272081	0,8307791	0,8061169	0,7724621	-0,7179551	-0,7080828	-0,4138376	0,9002104	-0,1234939	0,8857877	0,9002104
Eggy / custar	0,9635017	0,943394	-0,5526237	0,9743095	0,8406371	0,9994478	0,9970997	0,9914277	-0,9774264	-0,9743547	-0,8325645	0,9943329	0,419819	0,99723	0,9943329
Browned	-0,4776418	0,110888	0,9364073	-0,4386742	0,3385512	-0,192517	-0,1502104	-0,0957826	0,0140803	5,689E-16	-0,3523807	-0,3273268	0,7898653	-0,2968661	-0,3273268
Almond	-0,5982506	-0,0322255	0,9769336	-0,5625528	0,2006576	-0,3307273	-0,2899061	-0,2370005	0,1567789	0,1428571	-0,2150727	-0,4589569	0,6941525	-0,4302383	-0,4589569
Typical taste	0,956912	0,9509626	-0,5327904	0,9687201	0,8531911	0,9999537	0,9986188	0,9942363	-0,9821426	-0,9793962	-0,8454119	0,9915455	0,4411326	0,9951957	0,9915455
Intensity	0,8529273	0,4008846	-0,98641	0,8292261	0,1772801	0,6573715	0,6244131	0,5806513	-0,5121443	-0,5	-0,1627577	0,7559289	-0,3774033	0,7345532	0,7559289
Sweet taste	0,8785547	0,9938329	-0,3509149	0,8986462	0,940948	0,9812936	0,988654	0,9954023	-0,9999009	-1	-0,9358567	0,9449112	0,6132803	0,9549191	0,9449112
Bitter	-0,9004936	-0,9873557	0,3955103	-0,9186892	-0,9235911	-0,9894116	-0,9947304	-0,9988552	0,9994222	0,9988446	0,9178411	-0,9595498	-0,5746132	-0,9680823	-0,9595498
Carrot	0,1681747	0,701901	0,5186763	0,2112206	0,8480651	0,4557546	0,4935486	0,5404877	-0,6074651	-0,6185896	-0,8557818	0,3273268	0,9999773	0,357451	0,3273268
Metallic afte	-0,8529273	-0,4008846	0,98641	-0,8292261	-0,1772801	-0,6573715	-0,6244131	-0,5806513	0,5121443	0,5	0,1627577	-0,7559289	0,3774033	-0,7345532	-0,7559289
Sweet aftert	0,8893482	0,9910078	-0,372441	0,9085347	0,9328807	0,9854769	0,9918585	0,9973484	-0,9999594	-0,9997334	-0,9274716	0,9522166	0,5948806	0,9615186	0,9522166
Bitter afterta	-0,7302173	-0,9897827	0,1041708	-0,759462	-0,9958452	-0,9013523	-0,9191146	-0,9393324	0,9642313	0,9678678	0,9943951	-0,8322397	-0,7921934	-0,8495857	-0,8322397
Almond taste	-0,8312257	-0,3636878	0,9922235	-0,8060592	-0,1375263	-0,6265096	-0,59247	-0,5474132	0,4771607	0,4647394	0,1229149	-0,7289682	0,4143687	-0,7066479	-0,7289682
Adhesivenes	0,2140858	0,7344398	0,4781297	0,2566955	0,8719157	0,4968783	0,5336776	0,5792389	-0,6439451	-0,6546537	-0,8790368	0,3711537	0,9985683	0,4007316	0,3711537
Cohesivenes	0,7025706	0,1691019	-0,9969974	0,6707041	-0,0642975	0,4571263	0,4185208	0,3680999	-0,2908505	-0,2773501	0,0789965	0,5765567	-0,5887847	0,5500667	0,5765567
Dryness	-0,6164214	-0,9560661	-0,0500689	-0,6503429	-0,9979997	-0,8239983	-0,8475636	-0,8753836	0,911977	0,9176629	0,9988229	-0,7370435	-0,8766453	-0,7583312	-0,7370435
Springiness	0,9978701	0,8557457	-0,7112237	0,9997703	0,7122722	0,971584	0,9605263	0,9438303	-0,9136554	-0,9078413	-0,7018512	0,9950821	0,2255597	0,9913951	0,9950821
Chewiness	0,5220297	0,9161286	0,1643024	0,5589133	0,9841604	0,7535667	0,7810943	0,8141523	-0,8588994	-0,8660254	-0,9866661	0,6546537	0,926049	0,6785511	0,6546537

C

	Doughy / flo	Eggy / custar	Browned	Almond	Typical taste	Intensity	Sweet taste	Bitter	Carrot	Metallic afte	Sweet aftert	Bitter afterta	Almond taste	Adhesivenes	Cohesivenes	Dryness	Springiness	Chewiness
Weight	-0,7494737	-0,9861752	0,0604505	0,2024263	-0,989813	-0,5514372	-0,9981712	0,999923	-0,5699615	0,5514372	-0,9993008	0,950897	0,5174152	-0,6077602	-0,3349218	0,8919641	-0,9315288	-0,8342164
Volume	0,7933531	0,9952562	-0,1291563	-0,2694922	0,9972759	0,6076647	0,9916243	-0,9966854	0,5119285	-0,6076647	0,9943419	-0,9272837	-0,575208	0,5515375	0,3991164	-0,8586553	0,9543945	0,7941937
Hardness	-0,9179569	-0,9888965	0,3673141	0,4964177	-0,9851118	-0,7831518	-0,9300969	0,9466743	-0,2867443	0,7831518	-0,9383295	0,8078463	0,7574901	-0,331228	-0,6108665	0,7075597	-0,9984004	-0,6218305
Chewiness	-0,7736621	-0,9916731	0,0976655	0,238838	-0,9944374	-0,5821905	-0,9952193	0,9987629	-0,5388951	0,5821905	-0,9972089	0,9386818	0,5489953	-0,5776958	-0,3698582	0,8744675	-0,9444537	-0,8130524
Cohesivenes	0,6986022	0,971267	0,0133369	-0,1296443	0,9766157	0,4884054	0,9999111	-0,9981148	0,6290136	-0,4884054	0,9993366	-0,9711355	-0,4528889	0,6646772	0,2645117	-0,9228809	0,9021682	0,8726168
Springiness	-0,6628814	-0,9585066	-0,0620787	0,0811396	-0,9649705	-0,4452739	-0,9980713	0,9939348	-0,6661726	0,4452739	-0,9963719	0,9816114	0,4088756	-0,7003181	-0,2171718	0,9405606	-0,8800598	-0,8953944
ENERGY VAL	-0,7844075	-0,9937345	0,1147079	0,2554455	-0,9960965	-0,5960396	-0,9933993	0,997764	-0,5243788	0,5960396	-0,9957828	0,9326348	0,5632396	-0,5636215	-0,3857271	0,8660254	-0,9499475	-0,8029551
TPC	-0,7942077	-0,9953919	0,1305495	0,270845	-0,9973786	-0,6087801	-0,9914418	0,9965701	-0,510721	0,6087801	-0,9941916	0,9267567	0,5763568	-0,5503649	-0,4004043	0,8579343	-0,954813	-0,7933391
TFC	-0,9179943	-0,9888825	0,3674017	0,4964994	-0,9850956	-0,7832104	-0,9300623	0,946644	-0,2866541	0,7832104	-0,9382969	0,8077908	0,7575516	-0,3311391	-0,610941	0,7074931	-0,9984057	-0,6217568
FRAP	-0,9504297	-0,9711298	0,4509554	0,5738367	-0,9652262	-0,8368121	-0,8925465	0,9131868	-0,1977978	0,8368121	-0,9027202	0,75047	0,8140989	-0,2434186	-0,6808118	0,6398653	-0,9993824	-0,5474902
BETA 30	0,9524927	0,9695088	-0,4569283	-0,5793137	0,9634522	0,8404633	0,8895035	-0,9104344	0,1912225	-0,8404633	0,8998159	-0,7460229	-0,8179734	0,2369114	0,6857064	-0,6346995	0,9991244	0,5418685
BETA 60	0,9338326	0,9817691	-0,4061182	-0,5324986	0,9770072	0,808619	0,9138206	-0,9322815	0,246187	-0,808619	0,9229534	-0,7823353	-0,7842848	0,2912395	0,643634	-0,6772042	0,9998951	0,5883327
DPPH	0,9593664	0,9635017	-0,4776418	-0,5982506	0,956912	0,8529273	0,8785547	-0,9004936	0,1681747	-0,8529273	0,8893482	-0,7302173	-0,8312257	0,2140858	0,7025706	-0,6164214	0,9978701	0,5220297
ABTS	0,6254147	0,943394	0,110888	-0,0322255	0,9509626	0,4008846	0,9938329	-0,9873557	0,701901	-0,4008846	0,9910078	-0,9897827	-0,3636878	0,7344398	0,1691019	-0,9560661	0,8557457	0,9161286
AMYLASE	-0,9097016	-0,5526237	0,9364073	0,9769336	-0,5327904	-0,98641	-0,3509149	0,3955103	0,5186763	0,98641	-0,372441	0,1041708	0,9922235	0,4781297	-0,9969974	-0,0500689	-0,7112237	0,1643024
GLUCOSIDAS	0,9460766	0,9743095	-0,4386742	-0,5625528	0,9687201	0,8292261	0,8986462	-0,9186892	0,2112206	-0,8292261	0,9085347	-0,759462	-0,8060592	0,2566955	0,6707041	-0,6503429	0,9997703	0,5589133
L*(LUMINOS	0,4272081	0,8406371	0,3385512	0,2006576	0,8531911	0,1772801	0,940948	-0,9235911	0,8480651	-0,1772801	0,9328807	-0,9958452	-0,1375263	0,8719157	-0,0642975	-0,9979997	0,7122722	0,9841604
C* CHROMA	0,8307791	0,9994478	-0,192517	-0,3307273	0,9999537	0,6573715	0,9812936	-0,9894116	0,4557546	-0,6573715	0,9854769	-0,9013523	-0,6265096	0,4968783	0,4571263	-0,8239983	0,971584	0,7535667
General appe	0,8061169	0,9970997	-0,1502104	-0,2899061	0,9986188	0,6244131	0,988654	-0,9947304	0,4935486	-0,6244131	0,9918585	-0,9191146	-0,59247	0,5336776	0,4185208	-0,8475636	0,9605263	0,7810943
Glossy	0,7724621	0,9914277	-0,0957826	-0,2370005	0,9942363	0,5806513	0,9954023	-0,9988552	0,5404877	-0,5806513	0,9973484	-0,9393324	-0,5474132	0,5792389	0,3680999	-0,8753836	0,9438303	0,8141523
Embrowned	-0,7179551	-0,9774264	0,0140803	0,1567789	-0,9821426	-0,5121443	-0,9999009	0,9994222	-0,6074651	0,5121443	-0,9999594	0,9642313	0,4771607	-0,6439451	-0,2908505	0,911977	-0,9136554	-0,8588994
Arched	-0,7080828	-0,9743547	5,689E-16	0,1428571	-0,9793962	-0,5	-1	0,9988446	-0,6185896	0,5	-0,9997334	0,9678678	0,4647394	-0,6546537	-0,2773501	0,9176629	-0,9078413	-0,8660254
Open surface	-0,4138376	-0,8325645	-0,3523807	-0,2150727	-0,8454119	-0,1627577	-0,9358567	0,9178411	-0,8557818	0,1627577	-0,9274716	0,9943951	0,1229149	-0,8790368	0,0789965	0,9988229	-0,7018512	-0,9866661
Typical arom	0,9002104	0,9943329	-0,3273268	-0,4589569	0,9915455	0,7559289	0,9449112	-0,9595498	0,3273268	-0,7559289	0,9522166	-0,8322397	-0,7289682	0,3711537	0,5765567	-0,7370435	0,9950821	0,6546537
Sweet	-0,1234939	0,419819	0,7898653	0,6941525	0,4411326	-0,3774033	0,6132803	-0,5746132	0,9999773	0,3774033	0,5948806	-0,7921934	0,4143687	0,9985683	-0,5887847	-0,8766453	0,2255597	0,926049
Buttery	0,8857877	0,99723	-0,2968661	-0,4302383	0,9951957	0,7345532	0,9549191	-0,9680823	0,357451	-0,7345532	0,9615186	-0,8495857	-0,7066479	0,4007316	0,5500667	-0,7583312	0,9913951	0,6785511
Vanilla	0,9002104	0,9943329	-0,3273268	-0,4589569	0,9915455	0,7559289	0,9449112	-0,9595498	0,3273268	-0,7559289	0,9522166	-0,8322397	-0,7289682	0,3711537	0,5765567	-0,7370435	0,9950821	0,6546537
Doughy / flo	1	0,8488153	-0,7061294	-0,8000416	0,8360951	0,9655674	0,7080828	-0,7411991	-0,1168034	-0,9655674	0,724197	-0,5077676	-0,9543145	-0,0702347	0,874814	-0,3691939	0,9389167	0,260153
Eggy / custar	0,8488153	1	-0,2250176	-0,3619032	0,9997212	0,6820483	0,9743547	-0,9840426	0,4259261	-0,6820483	0,9792902	-0,8864638	-0,6520623	0,4677676	0,4864273	-0,8047163	0,9789124	0,7313071
Browned	-0,7061294	-0,2250176	1	0,9897433	-0,2019481	-0,8660254	3,206E-17	0,0480569	0,7857143	0,8660254	-0,0230879	-0,2514594	0,8854475	0,7559289	-0,9607689	-0,3973597	-0,4193139	0,5
Almond	-0,8000416	-0,3619032	0,9897433	1	-0,3397905	-0,9285714	-0,1428571	0,1902561	0,6892855	0,9285714	-0,1656701	-0,1106135	0,9427571	0,6546537	-0,9905361	-0,2621894	-0,5447048	0,3711537
Typical taste	0,8360951	0,9997212	-0,2019481	-0,3397905	1	0,6645903	0,9793962	-0,9879696	0,4471708	-0,6645903	0,9837977	-0,8971444	-0,6339785	0,4885069	0,4656611	-0,8185096	0,973816	0,747208
Intensity	0,9655674	0,6820483	-0,8660254	-0,9285714	0,6645903	1	0,5	-0,5410408	-0,3711537	-1	0,5198614	-0,2661637	-0,9991897	-0,3273268	0,9707253	-0,1147079	0,8170572	1,282E-15
Sweet taste	0,7080828	0,9743547	3,206E-17	-0,1428571	0,9793962	0,5	1	-0,9988446	0,6185896	-0,5	0,9997334	-0,9678678	-0,4647394	0,6546537	0,2773501	-0,9176629	0,9078413	0,8660254
Bitter	-0,7411991	-0,9840426	0,0480569	0,1902561	-0,9879696	-0,5410408	-0,9988446	1	-0,5801158	0,5410408	-0,9996879	0,9546652	0,5067543	-0,6175697	-0,3232013	0,8975068	-0,9269433	-0,8409963
Carrot	-0,1168034	0,4259261	0,7857143	0,6892855	0,4471708	-0,3711537	0,6185896	-0,5801158	1	0,3711537	0,6002842	-0,7962882	0,4082258	0,9989061	-0,583324	-0,8798679	0,2321202	0,9285714
Metallic afte	-0,9655674	-0,6820483	0,8660254	0,9285714	-0,6645903	-1	-0,5	0,5410408	0,3711537	1	-0,5198614	0,2661637	0,9991897	0,3273268	-0,9707253	0,1147079	-0,8170572	0
Sweet aftert	0,724197	0,9792902	-0,0230879	-0,1656701	0,9837977	0,5198614	0,9997334	-0,9996879	0,6002842	-0,5198614	1	-0,9618042	-0,4850586	0,6370264	0,2994583	-0,9082441	0,9172804	0,8542506
Bitter afterta	-0,5077676	-0,8864638	-0,2514594	-0,1106135	-0,8971444	-0,2661637	-0,9678678	0,9546652	-0,7962882	0,2661637	-0,9618042	1	0,2271522	-0,8237037	-0,0268438	0,9880963	-0,7732299	-0,9639279
Almond taste	-0,9543145	-0,6520623	0,8854475	0,9427571	-0,6339785	-0,9991897	-0,4647394	0,5067543	0,4082258	0,9991897	-0,4850586	0,2271522	1	0,365092	-0,979606	0,074633	-0,7931901	0,0402476
Adhesivenes	-0,0702347	0,4677676	0,7559289	0,6546537	0,4885069	-0,3273268	0,6546537	-0,6175697	0,9989061	0,3273268	0,6370264	-0,8237037	0,365092	1	-0,5447048	-0,9011271	0,2773501	0,9449112
Cohesivenes	0,874814	0,4864273	-0,9607689	-0,9905361	0,4656611	0,9707253	0,2773501	-0,3232013	-0,583324	-0,9707253	0,2994583	-0,0268438	-0,979606	-0,5447048	1	0,127257	0,6546537	-0,2401922
Dryness	-0,3691939	-0,8047163	-0,3973597	-0,2621894	-0,8185096	-0,1147079	-0,9176629	0,8975068	-0,8798679	0,1147079	-0,9082441	0,9880963	0,074633	-0,9011271	0,127257	1	-0,6664738	-0,9933993
Springiness	0,9389167	0,9789124	-0,4193139	-0,5447048	0,973816	0,8170572	0,9078413	-0,9269433	0,2321202	-0,8170572	0,9172804	-0,7732299	-0,7931901	0,2773501	0,6546537	-0,6664738	1	0,5765567
Chewiness	0,260153	0,7313071	0,5	0,3711537	0,747208	1,282E-15	0,8660254	-0,8409963	0,9285714	0	0,8542506	-0,9639279	0,0402476	0,9449112	-0,2401922	-0,9933993	0,5765567	1
